# Next‐generation sequencing for the diagnosis of *MYH9*‐RD: Predicting pathogenic variants

**DOI:** 10.1002/humu.23927

**Published:** 2019-10-15

**Authors:** Loredana Bury, Karyn Megy, Jonathan C. Stephens, Luigi Grassi, Daniel Greene, Nick Gleadall, Karina Althaus, David Allsup, Tadbir K. Bariana, Mariana Bonduel, Nora V. Butta, Peter Collins, Nicola Curry, Sri V. V. Deevi, Kate Downes, Daniel Duarte, Kim Elliott, Emanuela Falcinelli, Bruce Furie, David Keeling, Michele P. Lambert, Rachel Linger, Sarah Mangles, Rutendo Mapeta, Carolyn M. Millar, Christopher Penkett, David J. Perry, Kathleen E. Stirrups, Ernest Turro, Sarah K. Westbury, John Wu, NIHR BioResource, Keith Gomez, Kathleen Freson, Willem H. Ouwehand, Paolo Gresele, Ilenia Simeoni

**Affiliations:** ^1^ Department of Internal Medicine, Section of Internal and Cardiovascular Medicine University of Perugia Perugia Italy; ^2^ Department of Haematology University of Cambridge, Cambridge Biomedical Campus Cambridge UK; ^3^ NIHR BioResource ‐ Rare Diseases, Cambridge Biomedical Campus Cambridge University Hospitals Cambridge UK; ^4^ Department of Haematology, Addenbrooke's Hospital, Cambridge Biomedical Campus Cambridge University Hospitals NHS Foundation Trust Cambridge UK; ^5^ Institute for Immunology and Transfusion Medicine Universitätsmedizin Greifswald Ernst‐Moritz‐Arndt University Greifswald Greifswald Germany; ^6^ Transfusion Medicine Medical Faculty Tübingen Tübingen Germany; ^7^ Hull York Medical School University of Hull York UK; ^8^ The Katharine Dormandy Haemophilia Centre and Thrombosis Unit Royal Free London NHS Foundation Trust London UK; ^9^ Hematology/Oncology Department Hospital de Pediatría “Prof. Dr. Juan P. Garrahan” Buenos Aires Argentina; ^10^ Servicio de Hematología y Hemoterapia Hospital Universitario La Paz‐IDIPaz Madrid Spain; ^11^ Arthur Bloom Haemophilia Centre, Institute of Infection and Immunity, School of Medicine Cardiff University UK; ^12^ Department of Clinical Haematology, Oxford Haemophilia and Thrombosis Centre Oxford University Hospitals NHS Trust, Churchill Hospital Oxford UK; ^13^ Oxford Haemophilia & Thrombosis Centre, Department of Haematology, Oxford University Hospitals NHS Trust, Churchill Hospital, Oxford and the NIHR BRC, Blood Theme Oxford Centre for Haematology Oxford UK; ^14^ Beth Israel Deaconess Medical Center Harvard Medical School Boston Massachusetts; ^15^ Churchill Hospital Oxford University Hospitals UK; ^16^ Department of Pediatrics Perelman School of Medicine at the University of Pennsylvania Philadelphia Pennsylvania; ^17^ Division of Hematology Children's Hospital of Philadelphia Philadelphia Pennsylvania; ^18^ Basingstoke and Hampshire Hospital, NHS Foundation Trust UK; ^19^ Hampshire Hospital NHS Foundation Trust UK; ^20^ Centre for Haematology, Hammersmith Campus, Imperial College Academic Health Sciences Centre Imperial College London London UK; ^21^ Medical Research Council Biostatistics Unit, Cambridge Biomedical Campus Cambridge Institute of Public Health Cambridge UK; ^22^ School of Cellular and Molecular Medicine University of Bristol Bristol UK; ^23^ British Columbia Children's Hospital Vancouver Canada; ^24^ NIHR BioResource, Cambridge Biomedical Campus Cambridge University Hospitals Cambridge UK; ^25^ Department of Cardiovascular Sciences, Center for Molecular and Vascular Biology KU Leuven Leuven Belgium; ^26^ NHS Blood and Transplant Cambridge Biomedical Campus Cambridge UK; ^27^ Wellcome Trust Genome Campus Wellcome Trust Sanger Institute Cambridge UK

**Keywords:** ACMG guidelines, clinical diagnosis, genomics, high throughput sequencing, MYH9‐related disorders, variant classification

## Abstract

The heterogeneous manifestations of *MYH9*‐related disorder (MYH9‐RD), characterized by macrothrombocytopenia, Döhle‐like inclusion bodies in leukocytes, bleeding of variable severity with, in some cases, ear, eye, kidney, and liver involvement, make the diagnosis for these patients still challenging in clinical practice. We collected phenotypic data and analyzed the genetic variants in more than 3,000 patients with a bleeding or platelet disorder. Patients were enrolled in the BRIDGE‐BPD and ThromboGenomics Projects and their samples processed by high throughput sequencing (HTS). We identified 50 patients with a rare variant in *MYH9*. All patients had macrothrombocytes and all except two had thrombocytopenia. Some degree of bleeding diathesis was reported in 41 of the 50 patients. Eleven patients presented hearing impairment, three renal failure and two elevated liver enzymes. Among the 28 rare variants identified in *MYH9*, 12 were novel. HTS was instrumental in diagnosing 23 patients (46%). Our results confirm the clinical heterogeneity of *MYH9*‐RD and show that, in the presence of an unclassified platelet disorder with macrothrombocytes, *MYH9*‐RD should always be considered. A HTS‐based strategy is a reliable method to reach a conclusive diagnosis of *MYH9*‐RD in clinical practice.

## Graphical abstract

*MYH9*‐related disorder diagnosis is still challenging in clinical practice. We analyzed the genetic variants in more than 3,000 patients with a bleeding or platelet disorder and identified 50 patients with a rare variant in *MYH9*. In the presence of an unclassified platelet disorder with macrothrombocytes, *MYH9*‐RD should always be considered.

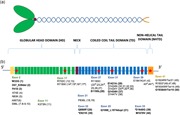

## 1 INTRODUCTION

Nonmuscle myosin heavy chain 9 related disorder (*MYH9*‐RD) is a rare autosomal‐dominant syndrome characterized by large/giant platelets and thrombocytopenia associated with the presence of Döhle‐like inclusion bodies in neutrophils (Kunishima et al., 2003). Clinical manifestations include a mild to moderate bleeding tendency (Orsini et al., 2017) and the risk of developing progressive nephropathy, sensorineural deafness, pre‐senile cataract, or alteration of liver enzymes during infancy or adult life (Balduini, Pecci, & Savoia, 2011; Pecci et al., 2012; Pecci, Ma, Savoia, & Adelstein, 2018). The disease is caused by heterozygous variants in *MYH9*, the gene coding for the heavy chain of nonmuscle myosin of class IIA (NMMHC‐IIA), a 1,960 amino acid residue protein involved in platelet cytoskeletal contraction, granule secretion, and in the Rho GTPases and Ca^2+^/calmodulin signaling pathways (Vicente‐Manzanares, Ma, Adelstein, & Horwitz, 2009). *MYH9* is located on chromosome 22q12‐13 and is composed of 41 exons. The coding region from exons 2–19 encodes for the globular head domain (HD), exon 20 for the neck region, and exons 21–40 for the coiled‐coil tail domain (TD). The final 34 amino acid residues of the C‐terminal nonhelical tail domain (NHTD) are encoded by exon 41.

About 101 *MYH9* variants are listed in the Human Gene Mutation Database (HGMD, public version, as of July 2019; Stenson et al., 2017): 72 missense/nonsense, 4 splicing substitutions, 25 deletions/insertions. Some cases of somatic or germinal mosaicism have also been described (Gresele et al., 2013; Kunishima et al., 2005; Kunishima, Takaki, Ito, & Saito, 2009).

Genotype–phenotype correlation studies in *MYH9*‐RD patients have reported that variants in the HD are associated with more severe thrombocytopenia and a higher frequency and/or a more rapid progression of nephropathy and deafness than variants in the TD, with the amino acid substitution p.Arg702Cys resulting in the most severe phenotype reported to date (Pecci et al., 2014; Pecci et al., 2008b; Saposnik et al., 2014). However, some exceptions exist: the p.Asp1424His variant which lies in the TD, is also associated with a high risk of developing syndromic manifestations. Moreover, patients carrying variants at the interface between the SH3‐like motif and the motor domain (MD) of the HD (SH3/MD interface), present a mild clinical phenotype consisting of mild macrothrombocytopenia and delayed risk of sensorineural deafness (Pecci et al., 2014).

The diagnosis of *MYH9*‐RD requires skilled laboratory investigations, including the correct assessment of the degree of thrombocytopenia, made difficult by the abnormal size of platelets, the identification of macrothrombocytes, and the determination of the presence of Döhle‐like inclusion bodies in neutrophils on a blood smear (Balduini et al., 2003). The latter test is performed by May–Grünwald–Giemsa (MGG) staining or through the identification of NMMHC‐IIA aggregates by immunofluorescence (Kunishima et al., 2003; Pecci et al., 2008b), a test which is not available in most of the hematology diagnostic laboratories despite its high sensitivity. Moreover, heterogeneity in the syndromic manifestations can complicate the interpretation of the clinical presentation. The identification of the causal *MYH9* variant in a patient is key to reach a conclusive diagnosis, predict the course of extra‐hematological symptoms and consequently implement a personalized clinical monitoring and therapeutic approach (Pecci et al., 2010a; Pecci, Granata, Fiore, & Balduini, 2008a). HTS techniques represent a comprehensive and cost‐effective strategy for diagnosing inherited bleeding, thrombotic and platelet disorders (BPDs; Simeoni et al., 2016; Zhang et al., 2016). The efficacy of HTS in patients with uncharacterized macrothrombocytopenia has been recently demonstrated (Rabbolini et al., 2017). Here, we report the patients with rare *MYH9* variants discovered after genome sequencing of 1,481 subjects enrolled in the BRIDGE‐BPD study and 1,550 patients enrolled in the clinical diagnostic ThromboGenomics study (Simeoni et al., 2016). We identified 28 causal rare *MYH9* variants in 50 patients (44 index cases), 20 with a diagnosis of *MYH9*‐RD based on the presence of macrothrombocytopenia, Döhle‐like bodies and an extra‐hematological phenotype in some cases but without genetic confirmation, 11 with suspected but unconfirmed *MYH9*‐RD, and 19 in whom *MYH9*‐RD was not previously suspected despite an expert evaluation of their clinical and laboratory data. We describe the 28 *MYH9* variants identified, 12 of which are novel, and classify the variants for pathogenicity and contribution to phenotype. We also describe the phenotypic profiles of this *MYH9*‐RD cohort, adding new insight into genotype–phenotype correlations and expanding the knowledge of this rare inherited platelet disorder.

## 2 METHODS

### 2.1 Patient cohort

Patients gave their written informed consent and were enrolled through two main projects: the NIHR BioResource ‐ Rare Diseases study (specifically, the BRIDGE‐BPD project) and the clinical diagnostic ThromboGenomics study. The BRIDGE‐BPD project includes patients with rare inherited BPDs of unknown etiology who were screened mainly by genome sequencing and a small subset by exome sequencing. DNA samples from BPD patients with clinical and laboratory phenotypes indicative of a particular molecular etiology were sequenced using the ThromboGenomics HTS test. Inclusion criteria have been previously described (Simeoni et al., 2016; Westbury et al., 2015). Ethics authorities and approval numbers are provided in Table S1.

### 2.2 Clinical and laboratory phenotypes

Clinical and laboratory phenotypes were submitted by the referring clinicians as Human Phenotype Ontology (HPO) terms, as previously described (Westbury et al., 2015). The severity of bleeding was coded as numerical scores using the MCMDM‐1 VWD Bleeding Assessment Tool (http://www1.wfh.org/docs/en/Resources/Assessment_Tools_MCMDM-1VWD.pdf). Centralized analysis of blood smears was performed by two independent centers for the identification of Döhle‐like inclusion bodies in patients who had either not been tested or had received an initial negative result for the presence of Döhle‐like inclusion bodies. Blood films obtained from patients and from healthy controls were randomly analyzed by two operators blindly. Inclusions in neutrophils were classified as type I, II, and III based on their size, shape and pattern of distribution (Kunishima et al., 2003; Pecci et al., 2008b). Information on hearing impairment, renal and liver dysfunctions were also collected.

### 2.3 Variant prioritization and assessment

Sequencing results were processed by using a single bioinformatic approach as previously described (Greene, BioResource, Richardson, & Turro, 2016; Simeoni et al., 2016; see also Supporting Information). An average of five variants per patient remained after bioinformatic filtering of variants and each of these variants was assessed following the ACMG Guidelines (Richards et al., 2015) by a MultiDisciplinary Team (MDT) composed of clinicians, clinical geneticists, bioinformaticians and clinical scientists. The Congenica software (Congenica Ltd., Hinxton, UK) was used to visualize the data and assign pathogenicity and contribution to phenotype to each variant based on the clinical picture, predicted consequence for the protein, presence in the Human Gene Mutation Database (HGMD; Stenson et al., 2017) and allele frequency in control datasets such as the Exome Aggregation Consortium (ExAC; Karczewski et al., 2017) and the genome Aggregation Database (gnomAD; Lek et al., 2016)). The MDT also evaluated the minor allele frequency (MAF) of the variants found in more than 13,000 participants enrolled in other non‐BPD BRIDGE projects. The LRG transcript LRG_567t1 (NM_002473.5, ENST00000216181.10) was used as the reference sequence. Variants and their pathogenicity have been deposited in ClinVar under accession numbers SCV000891130 to SCV000891157. They are accessible by searching for the accession number (e.g., SCV000891130) or with the keywords “MYH9 AND NIHR AND BioResource”(https://www.ncbi.nlm.nih.gov/clinvar/?term=MYH9+AND+NIHR+BioResource).

## 3 RESULTS

### 3.1 Novel *MYH9* variants

Total of 3,031 patients were enrolled in the BRIDGE‐BPD and ThromboGenomics studies and screened for rare variants in the *MYH9* gene. We found 74 individuals with a variant in the *MYH9* gene, however only 50 patients were considered for this study. The remaining 24 were excluded for the following reasons: (a) the *MYH9* variant was also present in other non‐BPD patients; (b) the platelet disorder and/or phenotype was not compatible with *MYH9*‐RD (e.g., thrombocytosis); (c) the phenotype was explained by the presence of a causal variant in another gene; (d) the *MYH9* variant was also identified in an unaffected family member. All the patients excluded from this study and the reasons for their exclusion are shown in detail in Table S2.

**Figure 1:**
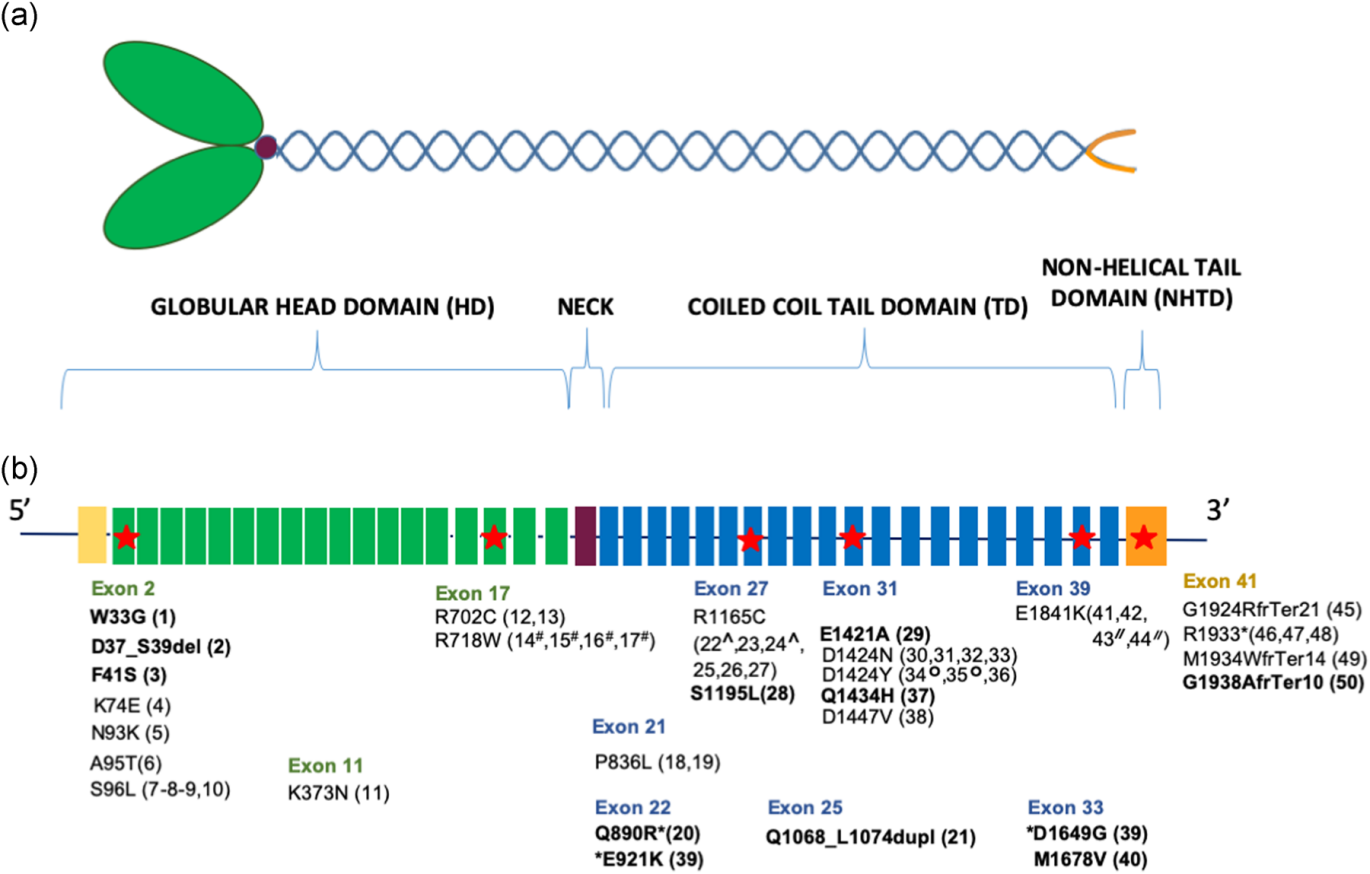
Schematic representation of the heavy chain A of nonmuscle myosin class IIA (NMMHC‐IIA) and variants position. (a) Schematic representation of NMMHC‐IIA protein. Nonmuscle myosin II A shows a hexameric structure consisting of two heavy chains, namely NMMHC‐IIA, and two pairs of light chains. Each heavy chain includes a N‐terminal globular head domain (HD), a neck region which binds the light chains, and a C‐terminal α‐helical coiled‐coil tail domain (TD), which ends with a nonhelical tail domain (NHTD) involved in the subcellular localization of the protein. The HD includes four subdomains: the N‐terminal SRC‐Homology 3 like motif (SH3), the upper and lower 50 kDa subdomains, that together form the motor domain (MD), and the converter subdomain. In green the globular HD, in violet the neck domain and in blue the coiled coil TD with the NHTD at the 3′‐UTR in orange. (b) Affected exons and variants identified. The most affected exons are highlighted with red stars. The novel variants are shown in bold and the number in brackets is the patient ID number. Colors reflect protein domains. All variants described were confirmed by Sanger sequencing. The * indicates the two mutations identified in the same patient (39). #*,^,°,^//^ represent members of the same family. 3′‐UTR, 3′‐untranslated region

In the remaining 50 patients analyzed, of whom 44 are index cases, we found 28 *MYH9* variants, namely 21 missense, three frameshifts, two stop gains, one in‐frame deletion, and one in‐frame insertion. The variants identified were positioned in 11 of the 41 exons of the *MYH9* gene (Figure 1). Of the 28 variants, 12 were absent from the HGMD database (public version, as of July 2019, Stenson et al., 2017), the literature and all other publicly accessible *MYH9*‐RD databases at the time of the analysis (Table 1). Of the novel variants, three affect the SH3/MD interface of the globular MYH9 head, including a new c.97T>G transversion in exon 2, leading to p.Trp33Gly amino acid change, an in‐frame deletion p.Asp37_Ser39del and one missense variant p.Phe41Ser caused by the c.353T>C transition. In silico protein modeling predicts that these three variants may disturb the hydrophobicity of the SH3/MD interface (Figure S1). We also found eight novel variants localized in the coiled‐coil domain. These include one missense variant p.Glu921Lys and a nonsense variant p.Gln890Arg*, leading to a premature stop codon causing the formation of a shorter MYH9 protein of 890 amino acids, both in exon 22; one in‐frame insertion p.Gln1068_Leu1074dup in exon 25 and five missense variants, p.Ser1195Leu in exon 27, p.Glu1421Ala and p.Gln1434His in exon 31, p.Asp1649Gly and p.Met1678Val in exon 33. In the nonhelical tail domain of the protein, we found one further novel variant and a frameshift leading to a premature stop in the protein, p.Gly1938Alafs*10. The read coverage of whole genome sequencing (WGS) and targeted sequencing results for the 12 novel variants (in 11 patients) are shown in Figure S2.

**Table 1:** MYH9 variants

Patient	Ethnicity	Chromosomal position	Transcript alteration	Protein alteration	MYH9 domain affected	Variant type	CADD score	AF (ExAC and gnomAD)	Pathogenicity and contribution to phenotype	ACMG	Diagnosis
		(NG_011884.2)	(NM_002473.5)	(NP_002464.1)						Evidence	
**Novel MYH9 variants**											
**1**	EUR	22:43879T**>**G	**c.97T>G**	**p.(Trp33Gly)**	**HD**	**Missense**	**27.20**	Not present	Likely pathogenic	PM2, PM5, PP3, PP4, A	New diagnosis MYH9‐RD not suspected
									Full		
**2**	EUR	22:43890_43898del	**c.108_116delCGACAAGAG**	**p.(Asp37_Ser39del)**	**HD**	**In‐frame deletion**	**N/A**	Not present	VUS	PM2, PM4, PP4, A	Known *MYH9‐RD* Patient with no genetic confirmation
									Full		
**3**	EUR	22:43904T**>**C	**c.122T>C**	**p.(Phe41Ser)**	**HD**	**Missense**	**28.7**	Not present	VUS	PM6, PM2, PP3, PP4, A	No diagnosis Assumed *de nov*o variant
									Full		
**20**	EUR	22:91998del	**c.2668delC**	p.(Gln890Arg*)	**TD**	**Stop‐gain**	**N/A**	Not present	^***^VUS	PM2, PVS1,	No diagnosis
									Full	PP4, A	
**39**	EUR	22:92091G**>**A	**c.2761 G>A**	**p.(Glu921Lys)**	**TD**	**Missense**	**33**	1:100,000 (ExAC and gnomAD)	VUS	PM2, A, B	No diagnosis
									Full		
**21**	EUR	22:96106_96126dup	**c.3202_3222dupCAGGCCCAGATCGCGGAGCTC**	**p.(Gln1068_Leu1074dup)**	**TD**	**In‐frame insertion**	**N/A**	Not present	VUS	PM2, PM4, PP4, A, B	Known *MYH9*‐RD Patient with no genetic confirmation
									Full		
**28**	EUR	22:98041C**>**T	**c.3584C>T**	**p.(Ser1195Leu)**	**TD**	**Missense**	**23.1**	Not present	VUS	PM2, A	No diagnosis
									Full		
**29**	EUR	22:100951A**>**C	**c.4262A>C**	**p.(Glu1421Ala)**	**TD**	**Missense**	**28.5**	Not present	VUS	PM2, PP3, A	No diagnosis
									Full		
**37**	AFR	22:100991G>C	**c.4302G>C**	**p.(Gln1434His)**	**TD**	**Missense**	**23.5**	1:10,000 (ExAC);1:250,000 (gnomAD)	VUS Full	PM2	No diagnosis
**39**	EUR	22:106186A**>**G	**c.4946A>G**	**p.(Asp1649Gly)**	**TD**	**Missense**	**32**	Not present	VUS	PM2, PP3, PP1, A, B	No diagnosis
									Full		
**40**	N/A	22:106272A**>**G	**c.5032A>G**	**p.(Met1678Val)**	**TD**	**Missense**	**18.4**	Not present	VUS	PM2, A	No diagnosis
									Uncertain		
**50**	N/A	22:110276del	**c.5808delG**	p.(Gly1938Alafs*10)	**NHTD**	**Frameshift**	**N/A**	Not present	Likely pathogenic	PM2, **#**PM4, PM1, PP4, A	Known *MYH9‐RD* Patient with no genetic confirmation
									Full		
**Known MYH9 variants**											
**4**	EUR	22:44002A**>**G	c.220A**>**G	p.(Lys74Glu; Kanematsu et al., 2016)	HD	Missense	23.50	Not present	Likely pathogenic Full	PS4_supporting, PM2, PP4, PP3, A, B	Known *MYH9*‐RD patient
**5**	EUR	22:.44061C**>**G	c.279C**>**G	p.(Asn93Lys; Seri et al., 2000)	HD	Missense	25	Not present	**Pathogenic Full	PS4, PS3, PM1, PM2, PP4, PP3, A	Known *MYH9*‐RD patient
**6**	EUR	22:.44065G**>**A	c.283G**>**A	p.(Ala95Thr; Kunishima et al., 2001b)	HD	Missense	29.70	Not present	**Pathogenic Full	PS4, PM6, PM2, PM1, PP3, A	New diagnosis *MYH9*‐RD suspected Assumed de novo variant
**7,8,9, 10**	EUR^7,8^,N/A^9^, Hispanic^10^	22:44069C**>**T	c.287C**>**T	p.(Ser96Lys; Arrondel et al., 2002)	HD	Missense	32.00	Not present	Pathogenic Full	PS4, PM1, PM2, PP4, PP3, A^8,10^	New diagnosis for patients 7, 8, and 9. Patients 7 and 9 (*MYH9*‐RD not suspected); 8 (suspected); and 10 (known *MYH9*‐RD).
**11**	EUR	22:74704G**>**C	c.1119G**>**C	p.(Lys373Asn; Arrondel et al., 2002)	HD	Missense	28.8	Not present	Likely pathogenic Full	PS4, PM2, PP4, PP3, A	New diagnosis *MYH9*‐RD suspected
**12, 13**	East Asian^12^, EUR^13^	22:87034C**>**T	c.2104C**>**T	p.(Arg702Cys; Seri et al., 2000)	HD	Missense	35	Not present	**Pathogenic Full	PS4, PS3, PM2, PP4, PP3, A^12^	New diagnosis for patient 13
											Patients 13 (*MYH9*‐RD suspected); 12 (known *MYH9*‐RD)
**14** ^#^ **,15** ^#^ **, 16** ^#^ **,17** ^#^	EUR^14,15,16,17^	22:87082C**>**T	c.2152C**>**T	p.(Arg718Trp; Pecci et al., 2008b)	HD	Missense	34	Not present in ExAC; 1:250,000 (gnomAD)	Pathogenic Full	PS4, PM2, PP1_strong, PP4, PP3, A^17^,B^14,15,16,17^	New diagnosis for all patients *MYH9*‐RD suspected
**18,19**	EUR^18,19^	22:91361C**>**T	c.2507C**>**T	p.(Pro836Lys; Neveling et al., 2013)	TD	Missense	34	Not present	*Likely pathogenic Full	PS4, PM2, PP4, PP3, A^18^	New diagnosis for both patients Patients 18 (MYH9‐RD suspected); 19 (not suspected)
**22**^**,23, 24**^**,25, 26,27**	EUR^22,23,24,26,27^ N/A^25^	22:97950C**>**T	c.3493C**>**T	p.(Arg1165Cys; Seri et al., 2000)	TD	Missense	34	Not present	Pathogenic Full	PS4, PM2, PP1_strong, PP4, PP3, A^25^, B^22–24^	New diagnosis for patients 23,25,26,27 Patients 23 and 25 (*MYH9*‐RD suspected); 26 and 27 (not suspected); 22 and 24 (known *MYH9*‐RD)
**30,31, 32,33**	Iraqui^30^, N/A^31^, SAS^32^, Hispanic^33^	22:100959G**>**A	c.4270G**>**A	p.(Asp1424Asn; Kunishima et al., 2001a)	TD	Missense	33	Not present	Pathogenic Full	PS4, PP1_strong, PM2, PP4, PP3, A^31,33^	New diagnosis for all patients
											Patients 31, 32, 33 (*MYH9*‐RD suspected); 30 (not suspected)
**34**°**,35**°**,36**	EUR^34,35,36^	22:100959G**>**T	c.4270G**>**T	p.(Asp1424Tyr; Kunishima et al., 2001b)	TD	Missense	32	Not present	Pathogenic Full	PS4, PP1_strong, PM2, PP4, PP3, A, B^34–35^	New diagnosis for patient 36
											Patient 36 (*MYH9*‐RD suspected); 34 and 35 (known *MYH9*‐RD)
**38**	EUR	22:101029A**>**T	c.4340A**>**T	p.(Asp1447Val; Pecci et al., 2008b)	TD	Missense	31	Not present	Likely pathogenic Full	PS4, PM2, PP4, PP3, A, B	Known *MYH9*‐RD patient with no genetic confirmation
**41,42, 43** ^//^ **, 44** ^//^	EUR^42,44,45^, Filipino^43^	22:108545G>A	c.5521G**>**A	p.(Glu1841Lys; Seri et al., 2000)	TD	Missense	34	Not present	Pathogenic Full	PS4, PP1_strong, PM2, PP4, PP3, A^41–44^, B^43,44^	Known *MYH9*‐RD Patients with no genetic confirmation
**45**	EUR	22:110238_110247del	c.5770_5779delGGGGACCTGC	p.(Gly1924Argfs*21; Pecci et al., 2010b)	NHTD	Frameshift	36	1:50,000 (ExAC); not present in gnomAD	Pathogenic Full	PS4, **#**PM4, PM2, PM1, PP4, A	New diagnosis *MYH9*‐RD not suspected
**46,47,48**	N/A	22:110265C**>**T	c.5797C**>**T	p.(Arg1933*; Seri et al., 2000)	NHTD	Stop‐gain	50	1:100,000 (ExAC); 1:250,000 (gnomAD)	Pathogenic Full	PS4, PM2, PM1, PP4, A^46–48^ B^47^	Known *MYH9*‐RD Patients with no genetic confirmation
**49**	EUR	22:110268del	c.5800delA	p.(Met1934Trpfs*14; Savoia et al., 2010)	NHTD	Frameshift	34	Not present	Likely pathogenic Full	PS4, **#**PM4, PM2, PM1, PP4, A	Known *MYH9*‐RD Patient with no
											genetic confirmation

*Note*: **ACMG Guidelines Evidence**: PVS1 (very strong evidence): Null variant (nonsense, frameshift, canonical ± 1 or 2 splice sites, initiation codon, single or multiexon deletion) in a gene where LOF is a known mechanism of disease; **PS1 (strong evidence):** same amino acid change as a previously published pathogenic variant regardless of nucleotide change; **PS4** (strong evidence): The prevalence of the variant in affected individuals is significantly increased compared with the prevalence in controls; **PS3** (strong evidence): Well‐established in vitro or in vivo functional studies supportive of a damaging effect on the gene or gene product; **PM2** (moderate evidence): Absent from controls (or at extremely low frequency if recessive) in Exome Sequencing Project, 1000 Genomes Project, or Exome Aggregation Consortium; **PM1** (moderate evidence): Located in a mutational hot spot and/or critical and well‐established functional domain (e.g., active site of an enzyme) without benign variation; **PM4** (moderate evidence): Protein length changes as a result of in‐frame deletions/insertions in a non repeat region or stop‐loss variants; **PM6** (supporting evidence): Assumed de novo, but without confirmation of paternity and maternity; **PP2** (supporting evidence): Missense variant in a gene that has a low rate of benign missense variation and in which missense variants are a common mechanism of disease; **PP3** (supporting evidence): Multiple lines of computational evidence support a deleterious effect on the gene or gene product (conservation, evolutionary, splicing impact, etc.); **PP1** (supporting evidence): Cosegregation with disease in multiple affected family members in a gene definitively known to cause the disease; **PP4** (supporting evidence): Patient's phenotype or family history is highly specific for a disease with a single genetic etiology; **#PM4** used instead of PVS1 in case of protein termination within the last 50 amino acids; **A**: presence of at least one main *MYH9*‐RD feature in addition to macrothrombocytes (Dohle‐like bodies, hearing impairment, nephropathy, and alteration of liver enzymes); **B**: family history of *MYH9*‐RD.

The ACMG evidence PP3 is used only in case of all the following evidence are present: highly conserved nucleotide, highly conserved amino acid, effect on protein, alignment with GVGD of C65 and deleterious prediction in SIFT, Mutation Taster and PolyPhen.

Superscript numbers in the ‘Ethnicity’ and in the ‘ACMG Evidence’ columns represent the patients. *Variant initially considered as ‘VUS’ but then changed into ‘likely pathogenic’; **Variants initially labeled as ‘likely pathogenic’ and subsequently re‐classified as ‘pathogenic’; ***variant labeled as VUS, independently by the ACMG evidence, due to the impossibility to perform functional tests.

Members of the same family are represented with the following symbols: #,^,°,^//^

Abbreviations: AFR, African; ACMG, American College of Medical Genetics; CADD, combined annotation dependent depletion; EUR, European; ExAC, exome aggregation consortium; gnomAD, Genome Aggregation Database; HD, head domain; MDT, multidisciplinary team; NHTD, nonhelical tail domain; SAS, South Asian; TD, coiled‐coil tail domain; VUS, variant of uncertain significance.

List of variants with nucleotide change position, protein alteration and allele frequency. Cases labeled from 1 to 50. Ethnicity indicated when known. MTD outcome is shown per each variant as pathogenicity and contribution to phenotype.

### 3.2 Variant pathogenicity and contribution to phenotype

The MDT assigned pathogenicity and contribution to phenotype to each variant according to the clinical features of each patient following the ACMG Standards and Guidelines (Richards et al., 2015; shown in Table 1). The choice of the transcript for variant reporting was based on transcript and protein lengths, and expression in blood cells according to the Blueprint data (Javierre et al., 2016). Eleven *MYH9* transcripts are expressed in the different blood cells, but only three of them are protein coding. ENST00000216181 (NM_002473, LRG_567t1) is the longest transcripts (7,501 base pairs (bp), corresponding to a protein with the expected 1,960 amino acids (aa) length), with an equivalent in the RefSeq database (NM_0024736). This is the most expressed transcript in platelets, while its expression is lower in neutrophils and megakaryocytes and much lower in erythroblasts. Of the two remaining protein coding transcripts, ENST00000401701 is much shorter (789 bp, 218aa) and markedly less expressed; ENST00000456729 is also shorter (449 bp, 103aa) and absent (log2(FPKM) < 1) in blood cells (Figure S3). For these reasons, ENST00000216181 (NM_002473) was used for variant reporting. This is also the transcript subsequently selected by LRG (LRG_567t1).

The MDT classified the novel variants as follows: the three variants in exon 2 found in patients 1, 2, and 3, as likely pathogenic (in patient 1) and variant of uncertain significance (VUS; in patients 2 and 3) with full contribution to phenotype. The stop gain p.Gln890Arg*, in patient 20, was classified as VUS with full contribution to the phenotype. Based on the high impact of the variant on the MYH9 protein causing a premature stop, the variant might be considered to be likely pathogenic. However, we have not been able to perform any functional tests due to the difficulties of recalling the 86‐year‐old patient, thus we remained conservative and classified this variant as VUS. The in‐frame insertion Gln1068_Leu1074dup and the frameshift variant, p.Gly1938Alafs*10, were considered VUS and likely pathogenic with full contribution to the phenotype, respectively. The six novel missense variants (present in patients 28‐29‐37‐39‐40) identified in the coiled‐coil domain of the MYH9 protein were classified as VUSs with full contribution to phenotype. The referring clinicians of these five patients with a VUS variant, were re‐contacted to arrange cosegregation studies. Pedigree analysis was possible only for two of these patients. This has confirmed, in patient 28, the absence of the variant in the nonaffected mother and in patient 39 the presence of the same variant in the daughter affected with mild thrombocytopenia. The pathogenicity and contribution to phenotype assigned to the remaining nonnovel variants are listed in Table 1.

**Figure 2:**
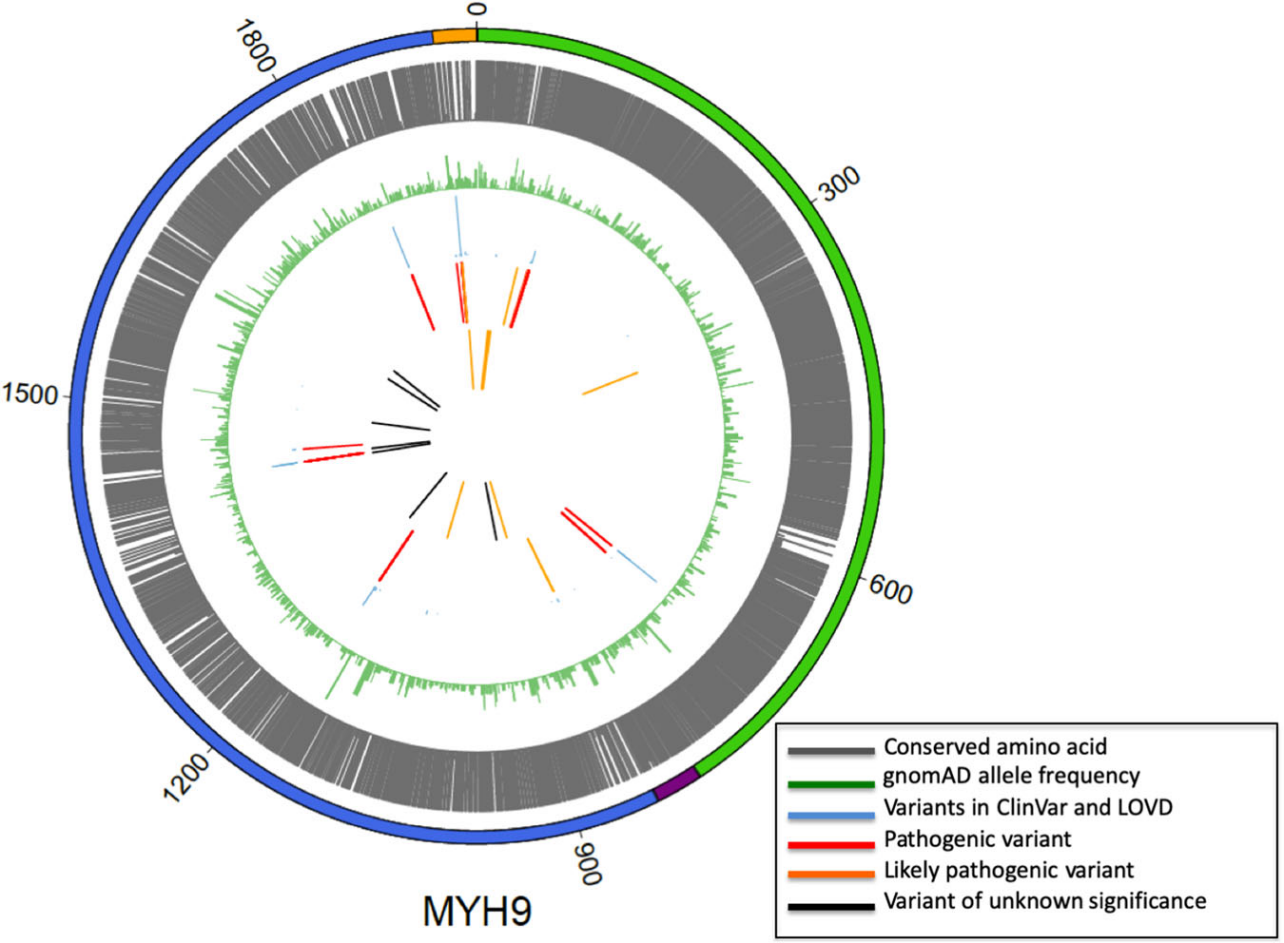
Evolutionary conservation variant analysis. From the outer to the inner circle. MYH9 protein domains: in green the N‐terminal globular head domain (HD), in purple the neck domain, in blue the C‐terminal α‐helical coiled‐coil tail domain (TD) and in orange the 3′‐UTR. Evolutionary conserved regions in the MYH9 protein in gray. All the pathogenic, likely pathogenic and VUS variants affect highly conserved amino acid residues. Variant minor allele frequency (MAF) in gnomAD database is represented by green bars. Smaller is the green bar lower is the allele frequency. Variants present in ClinVar and LOVD are represented by blue bars. The height of each blue bar represents the number of patients previously described with the same variant. Variants in this cohort previously seen in the literature include ‘pathogenic’ and ‘likely pathogenic’ variants, in red and orange, respectively. Novel variants in this cohort include ‘likely pathogenic” variants and VUS in orange and black, respectively. 3′‐UTR, 3′‐untranslated region

For all the variants identified in this study we investigated the evolutionary conservation in the MYH9 protein domains. We found that all pathogenic, likely pathogenic and VUS variants affect highly conserved amino acid residues providing further confidence that the variants identified have an impact on MYH9 protein function and consequently on the patients’ phenotypes (Figure 2).

### 3.3 Immunofluorescence analysis

At enrollment, the presence of Döhle‐like inclusion bodies was reported only in 21 (42%) of the 50 patients analyzed. Given that the Döhle‐like bodies are reported to be invariably present in *MYH9*‐RD patients, at least when analyzed by immunofluorescence, we recalled the remaining 29 patients, initially labeled as Döhle‐like bodies negative, for a centralized blood smear analysis (Table S3). Of these 29 patients, we obtained a fresh blood smear from 18 patients. An abnormal neutrophils MYH9 distribution was found in all 18 (100%) patients when analyzed by immunofluorescence and in 11 patients (61%) when analyzed by the MGG staining, in accordance with previous results (Balduini et al., 2011).

Immunofluorescence was performed for all the 11 patients with novel variants, except for those in which the Döhle‐like bodies were previously identified (patients 2, 3, 21, and 50) and in patients 37 and 39 not available for further analysis. In the remaining five patients, we obtained the following results: in patients 1 and 20, neutrophils had circular to oval shaped cytoplasmic spots that have been classified as type II inclusions. In patients 28 and 40, neutrophils had speckled inclusions and in patient 29 inclusions resembled small dots scattered throughout the cytoplasm inclusions were classified as type III.

**Figure 3:**
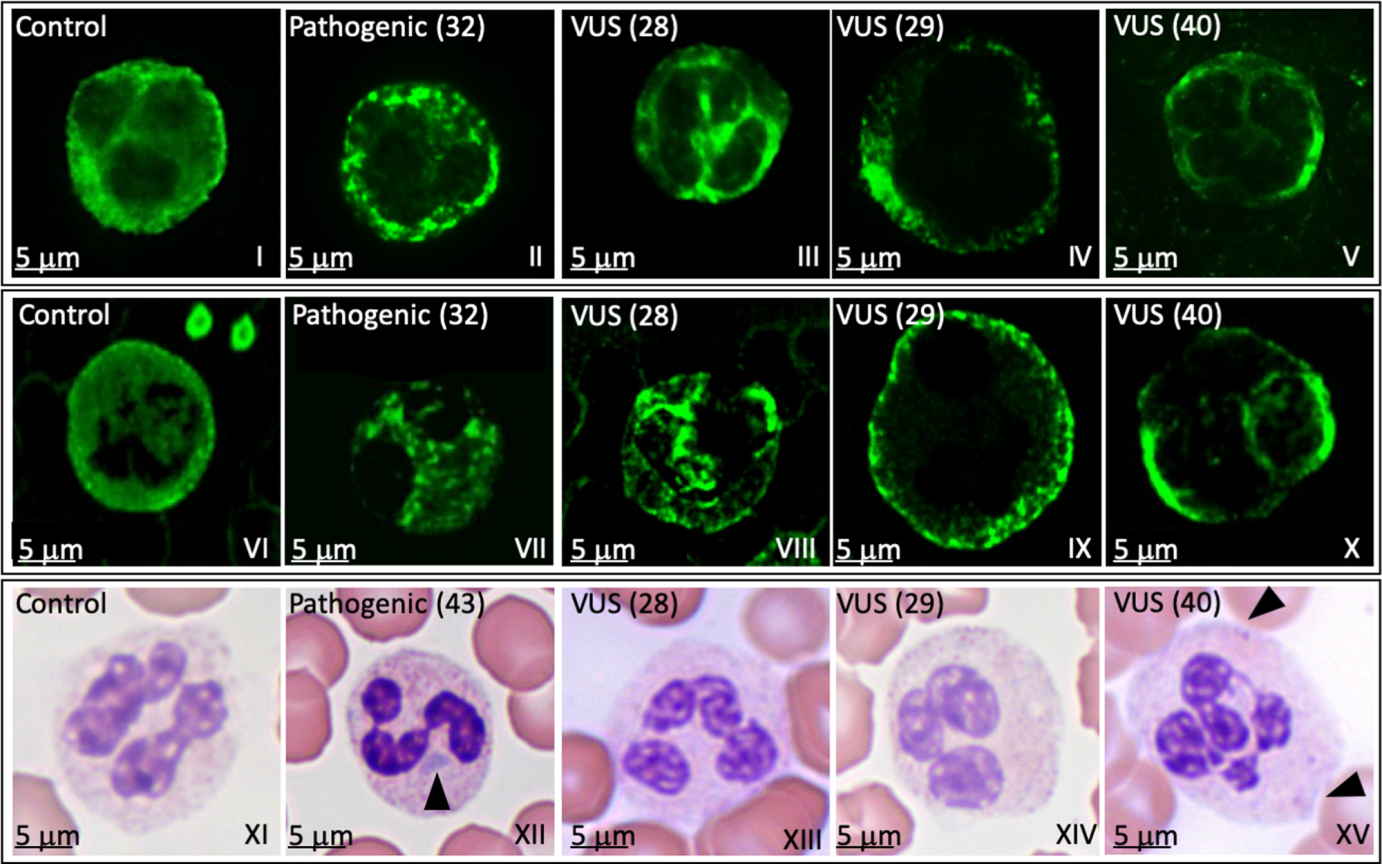
Döhle‐like inclusion bodies localization by NMMHC‐IIA immunofluorescence or MGG staining. Light microscopy and immunofluorescence analyses of granulocytes in a healthy control (control), in patients (32 for immunofluorescence and 43 for light microscopy) with a pathogenic variant (pathogenic) and in three patients (28, 29, and 40) with a variant of uncertain significance (VUS). The analysis was performed by two independent centres: Panels I–V show results obtained by centre 1; Panels VI–X show results obtained by centre 2. Both centres used rabbit antihuman NMMHCIIA Ab followed by Alexa‐Fluor 488‐conjugated secondary antibody. Results between the two centers were highly comparable. The patient's sample in which a pathogenic variant was identified shows circular to oval shaped cytoplasmic punctuate spots, classified as type II inclusions (panels II and VII). Patients’ samples in which VUSs were identified show a speckled staining (panels III and VIII and panels V and X, respectively), and many small dots scattered throughout the cytoplasm (panels IV and IX) classified as type III inclusions. Panels XI–XV show May–Grünwald–Giemsa staining. Panels XII and XV show the presence of Döhle‐like bodies (arrowhead) in patients’ samples with a pathogenic variant (XII) and a VUS (XV). NMMHCIIA, nonmuscle myosin of class IIA; VUS, variant of uncertain significance

In conclusion, 33% of the all the 18 patients re‐analyzed had type II inclusions and 67% type III myosin IIA inclusions (Table S3). An example of the altered NMMHC‐IIA distribution in neutrophils in patients with a pathogenic variant and VUS is shown in Figure 3 **.**

### 3.4 Phenotypic description of the *MYH9*‐RD cohort and genotype–phenotype correlation

Our cohort includes 21 males and 29 females from 44 unrelated pedigrees. The median age at diagnosis was 20 years (range 1‐76). Over a third (19) of the patients were enrolled with a diagnosis of ‘unclassified platelet disorder’ while the remaining (31) had a suspected (11) or known (20) but unconfirmed *MYH9*‐RD, based on family history, presence of large/giant platelets, thrombocytopenia, presence of Döhle‐like bodies and/or extra‐hematological symptoms.

**Figure 4:**
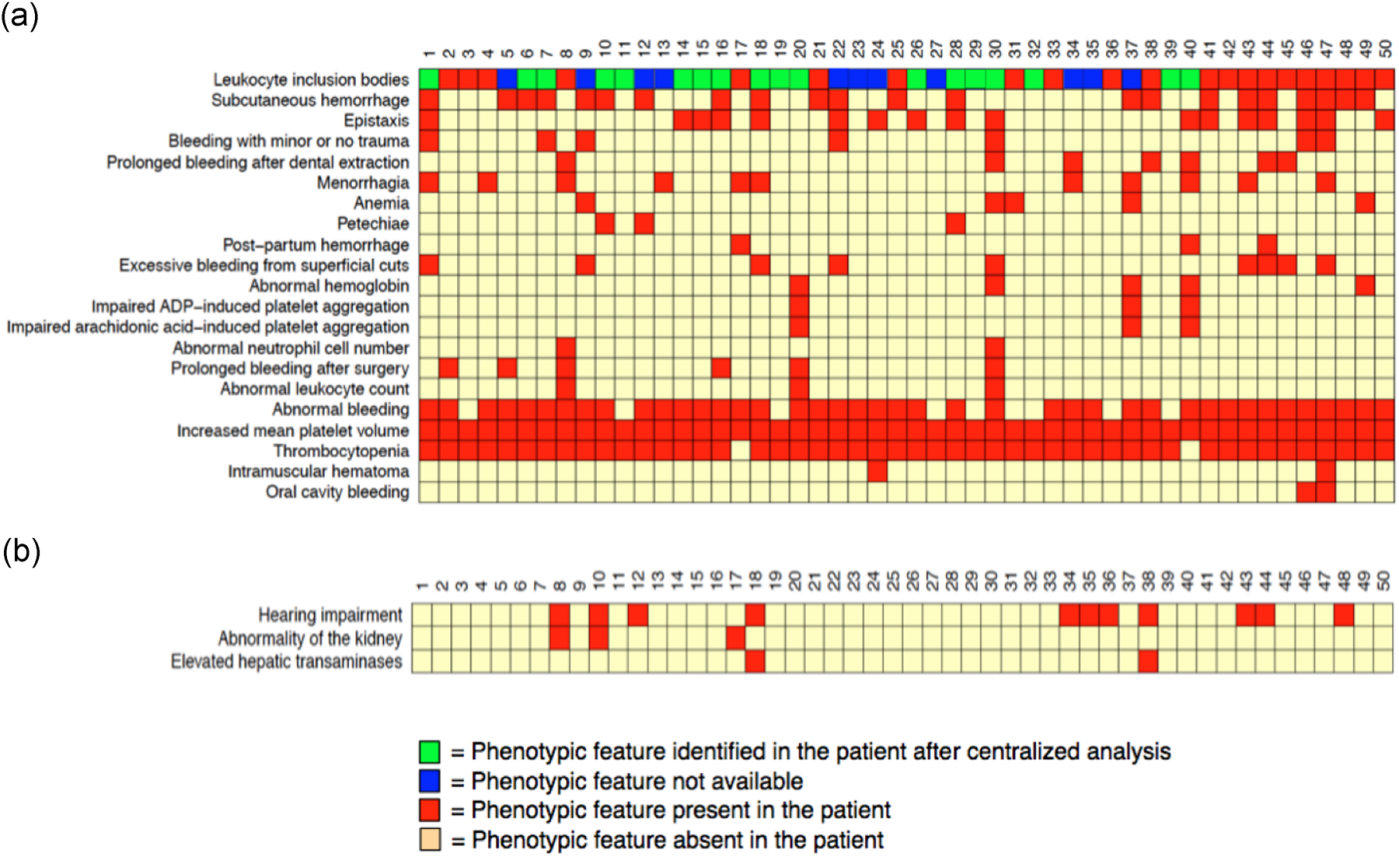
Cohort Phenotype. (a) HPO terms coded for hematological and (b) nonhematological symptoms. Y axis: HPO terms; X axis: patient ID number (from 1 to 50). Red box: the presence of the phenotypic feature; green box: the presence of NMMHC‐IIA aggregates identified only after centralized immunofluorescence analysis; and blue box: data not available. Pale yellow box: the absence of phenotypic feature. NMMHCIIA, nonmuscle myosin of class IIA

Macrothrombocytes were present in all patients, while thrombocytopenia, with various degrees of severity, was present in all but two patients (17 and 40). The median platelet count was 54 × 10^9^/L (8‐220 × 10^9^/L) from automated measurements and 48.5 × 10^9^/L by microscopic assessment, although the latter was only available for eight patients (Table S4). The mean platelet volume (MPV) values are shown in Table S5. Three cases had a normal MPV when measured by automatic blood cell counting, however, macrothrombocytes were noticed upon examination of their blood smears (Greinacher et al., 2017; Kunishima et al., 2001a). Hematological and non hematological symptoms are shown in Figure 4. Bleeding symptoms, mostly mild mucocutaneous bleeding, were reported in 82% of the patients (41 out of 50). Bleeding scores, calculated by the MCMDM‐1 VWD Bleeding Assessment Tool, are shown in Table S6. Of the 29 females enrolled, 11 (38%) had menorrhagia, one of the most common symptoms reported by women with congenital platelet disorders.

Genotype–phenotype correlations were analyzed by plotting the seven HPO terms representing the major *MYH9*‐RD clinical features against the exons in which both previously described and novel *MYH9* variants were found (Figure S4). We first investigated the correlation between the position of variants in the MYH9 protein and the degree of thrombocytopenia, by dividing patients into two groups according to the platelet count being below (severe/moderate) or above 50 × 10^9^/L (mild). We found that 39% of the patients with severe thrombocytopenia have a variant affecting exons in the HD and 61% of the individuals had a variant in the coiled coil domain instead. Genotype–phenotype correlations were also studied for the extra‐hematological manifestations of *MYH9*‐RD. Details on how patients were screened for hearing impairment, renal dysfunction, and liver enzymes alteration are summarized in Table S7. Nephropathy was reported in patients 8 and 17, who carry p.Ser96Leu and p.Arg718Trp variants, respectively. Patient 8 also has hearing impairment. However, five other patients (10% of this cohort), two unrelated individuals carrying the same variant, p.Ser96Leu (patients 7 and 9), and three pedigree members of case 17, carrying the p.Arg718Trp, did not present any of these nonhematological features. Hearing impairment was present in 22% of the patients: 8% with variants involving the HD, as expected, and 14% involving the coiled‐coil and the NHT domains (Balduini, Pecci, & Noris, 2012; Pecci et al., 2014, 2008b). Variants observed in patients with bleeding symptoms were randomly distributed across the MYH9 domains, confirming a lack of genotype–phenotype correlation for the bleeding phenotype (Pecci et al., 2014; Saposnik et al., 2014). Moreover, no correlation was found even between platelet count and bleeding tendency (Figure S5).

## 4 DISCUSSION

*MYH9*‐RD, although rare, is considered the most frequent inherited macrothrombocytopenia. In Italy, where a large active patient registry was established in 2006, *MYH9*‐RD has an estimated frequency of 1 in 312,000, representing 12% of the inherited thrombocytopenias (Balduini et al., 2012; Pecci et al., 2014). The complexity and variability of patients’ phenotypes can make the diagnosis of *MYH9*‐RD rather challenging, even by skilled clinicians at specialist centres. As a consequence, a significant number of patients with *MYH9*‐RD are initially misdiagnosed as immune thrombocytopenic purpura (ITP), and thus subjected to ineffective and potentially harmful treatments, or classified as inherited platelet disorder of unknown origin. In this scenario, HTS techniques may represent a reliable method for the diagnosis of *MYH9*‐RD.

The present study represents the first systematic analysis of *MYH9* variants by HTS analysis in a large cohort of patients and controls enrolled from over 100 centres worldwide. Here, we report 50 *MYH9*‐RD patients with 28 rare variants in *MYH9* found in a group of 3,031 patients (of whom 764 were classified as having thrombocytopenia) and over 13,000 controls.

In agreement with previous studies, 75% of the variants identified (21 out of 28) are annotated in the most commonly affected *MYH9* exons (Pecci et al., 2014, 2008b; Saposnik et al., 2014).

We identified 12 novel variants affecting *MYH9* in highly conserved amino acid residues, including eight missense variants (one in a previously described amino acid residue but with a different nucleotide change (Jang et al., 2012; Kahr et al., 2009), one in‐frame deletion, one stop gain, one in‐frame insertion, and one frameshift. Patients carrying the eight missense variants did not show extra‐hematological symptoms, had a platelet count ranging from 15 to 96 × 10^9^/L and three of them (1, 20, and 21) had a history of excessive bleeding. The in‐frame deletion, Asp37_Ser39del**,** was found in a 31‐year‐old man with no bleeding symptoms, moderate thrombocytopenia and no extra‐hematological symptoms. The new pathogenic stop codon in the coiled‐coil domain, Gln890Arg*, leading to removal of 1,070 amino acids in the MYH9 protein was found in an 86‐year‐old man who was originally diagnosed as having an “unclassified platelet disorder”, with mild thrombocytopenia (88 × 10^9^/L), no extra‐hematological manifestations and a pathologic bleeding score due to major bleeding after surgery. We have not been able to test the presence of the truncated MYH9 protein in this patient's cells, and a non classical distribution of NMMHC‐IIA, with just small punctuate clusters (Althaus & Greinacher, 2009) was observed by IF‐ and MGG‐staining in granulocytes (Figure S6). We also report the first in‐frame insertion, Gln1068_Leu1074dup. The same amino acids were previously described to be involved in an in‐frame deletion in two patients (Ishida, Mori, Ota, Inaba, & Kunishima, 2013; Saposnik et al., 2014). This was found in a young girl who presented with moderate thrombocytopenia (70 × 10^9^/L), large platelets and moderate/severe bleeding (Bleeding score 7), similarly to the previously published cases, but with no current extra‐hematological symptoms. In contrast, the two patients previously described present several extra‐hematological features like hearing loss since childhood, congenital cataracts and mild proteinuria in a 59 year‐old woman (Saposnik et al., 2014) and end‐stage renal disease and bilateral hearing loss in a 27‐year‐old woman (Ishida et al., 2013). The novel frameshift, Gly1938Alafs*10, located in a known mutational hot spot, was found in a patient with severe thrombocytopenia (16 × 10^9^/L), large platelets and mild bleeding.

All the variants were discussed in MDT meetings and pathogenicity and contribution to phenotype assigned according to the ACMG Guidelines. The novel variants were labeled as pathogenic or likely pathogenic when supported by strong evidence, including the impact of the variant on the protein, the presence of strong *MYH9*‐RD phenotype and/or another *MYH9*‐RD feature and, when possible, by pedigree analysis. In all the remaining cases, the novel variants were classified as VUS. Previously reported variants were classified mainly as pathogenic. One variant, initially classified as VUS, was re‐classified as benign (patient 71 in Table S2), one variant initially VUS to a likely pathogenic (in patients 18 and 19) and four initially likely pathogenic as pathogenic (in patients 5, 6, 12, and 13).

We have previously shown that HTS technologies can successfully be applied to diagnose inherited bleeding, platelet and thrombotic disorders (Simeoni et al., 2016). In the present study, a total of 23 patients, 12/19 initially coded as “unclassified platelet disorder” and 11/11 for whom only a suspicion of *MYH9*‐RD was put forward with no conclusive diagnosis, received a molecular diagnosis of *MYH9*‐RD because a likely pathogenic or pathogenic variant in *MYH9* was found.

Our data confirm that the presence of Döhle‐like bodies is an invariable feature of *MYH9*‐RD. Indeed, Döhle‐like bodies were found in all 18 patients that were re‐analyzed by immunofluorescence (in 9 patients with a pathogenic, in 5 patients with a likely pathogenic, and in 4 patients with a VUS variant) and in 11/18 by MGG (in 6 patients with a pathogenic, in 4 patients with a likely pathogenic, and in a single patient with a VUS variant) bringing the percentage of patients positive for Döhle‐like bodies inclusion and with a variant in the MYH9 to 100%. Interestingly, we noted that 52% (11 out of the 21) of the patients in which Döhle‐like bodies were reported at enrollment by MGG staining had a variant in the tail or in the S2 fragment, which are the regions of the MYH9 protein that, when mutated, are associated with the presence of type I inclusions, the most visible at MGG staining and more easy to identify. Our attempt to identify genotype/phenotype correlations in this cohort of patients generally confirms previously published data (Pecci et al., 2014, 2008b; Saposnik et al., 2014), although with some exceptions. Our study confirms that variants in the HD are frequently associated with more severe thrombocytopenia and higher risk of other organ involvement contrarily to variants in the TD. In fact, two patients (cases 8 and 10) with severe/moderate thrombocytopenia, kidney disease and hearing impairment had variants in the HD (exon 2 and 17, respectively), while most of the cases with variants in the TD showed mild thrombocytopenia and no extra‐hematological organ involvement. Regarding the exceptions, three patients in our series carrying variants in the HD (patients 11, 12, and 13) had only mild thrombocytopenia, very mild or absent bleeding symptoms, and no other extra‐hematological manifestations, except hearing loss in patient 12. Moreover, four patients in our cohort (cases 28, 30, 33, and 50) with variants in the TD had severe thrombocytopenia (≤20 × 10^9^/L). Also patients carrying the same variant (p.Ser96Leu and p.Arg718Trp) did not share the same extra‐hematological phenotype, showing that the risk of developing deafness or renal failure may be variable among patients carrying the same variant. The risk to develop these phenotypes is known to increase with age. In our cohort we did not observe a clear age‐dependency for the development of an extra‐hematological phenotype, however, it must be considered that our patients are mostly relatively young. For instance, patients 12 and 13 with the p.Arg702Cys variant are 7 and 11 years old, respectively. Their health care management will take in consideration the high risk of developing extra‐hematologic features by age of 40 due to this known pathogenic variant.

Our cohort confirms that the presence of macrothrombocytes is an invariable feature of this disorder while thrombocytopenia, although highly frequent, may be absent (Pecci et al., 2014; Saposnik et al., 2014). This is the case for patients 17 and 40, with a platelet count of 187 and 220 × 10^9^ /L, respectively. Patient 17 shares the same variant with her sister (patient 14), her nephew (patient 15) and her mother (patient 16) who have all three mild thrombocytopenia. Unfortunately, we could not investigate further patient 40. Thus, this cohort confirms that a wide platelet count variability is a feature of *MYH9*‐RD (Balduini et al., 2011). In conclusion, our study expands the number of variants causing *MYH9*‐RD, highlights the heterogeneity of the *MYH9*‐RD phenotypes and, despite supporting previous correlation studies, shows that exceptions exist in genotype/phenotype correlations. The application of HTS‐based strategies revealed to be a reliable and fast method to reach a conclusive diagnosis of *MYH9*‐RD and exclude other thrombocytopenias with potential susceptibility to malignancies and may represent the first line of investigation for this disorder, even after preliminary expert evaluation.

## AUTHOR CONTRIBUTIONS

L. B. wrote the paper, performed immunofluorescence and provided samples; K. M. reviewed the paper, managed data, set up, and oversaw MDT meetings; J. C. S. provided genotyping results and processed samples; L. G. provided BLUEPRINT data; D. G. performed statistical analysis and provided HPO tables; N. G. performed variant conservation analysis; K. A. performed immunofluorescence; D. A., T. K. B, M. B., N. V. B., P. C., N. C., K. E., E. F, B. F., D. K., C. M. M., M. P. L, S. M., D. J. P., S. S, S. K. W., K. F., K. G., L. B., and P. G. provided samples and clinical data; S. V. V. D supported the bioinformatics analysis, K. D. managed ThromboGenomics; R. M. performed DNA extraction; D. D processed ThromboGenomics samples, S. P. coordinated the NIHR BioResource Rare Diseases BPD project; C. P. managed and supervised the WGS pipeline; K. S. managed the NIHR BioResource sequencing pipeline; E. T. managed BRIDGE‐BPD data analysis; K. F. and K. G. chaired the MDT meetings; P. G., K. F., and W. H. O. reviewed the paper; I. S. wrote the paper, managed, and processed ThromboGenomics samples.

## FUNDING INFORMATION

WHO is supported by: NIHR RG65966 and RBAG/181, NIHR BioResource ‐ Rare Diseases, British Heart Foundation (RBAG/245, 208, 226), European Commission (RBAG/344), MRC (RBAG/285, 295), NHS Blood and Transplant (RBAG/142), and Wellcome Trust (RBAG/342). P. G. is supported by: MIUR‐FIRB (Protocol #RBFR12W5V5_004), Telethon Foundation Grant (GGP15063), and Fondazione Umberto Veronesi. C. M. M. is supported by the NIHR Imperial College Biomedical Research Centre. N. V. B. is recipient of FIS‐Fondos FEDER CP14/00024.

## DATA AVAILABILITY STATEMENT

The data that support the findings of this study are available from the corresponding author upon reasonable request.

## Supporting information

Supporting information
