# Supplementary material for: Next‐generation sequencing for the diagnosis of MYH9‐RD: Predicting pathogenic variants
Source: Hum Mutat. 2019 Oct 15;41(1):277–90. doi: 10.1002/humu.23927 (PMC6972977; doi:10.1002/humu.23927)

Supporting Information for

Next-generation sequencing for the diagnosis of MYH9-RD

Supplemental methods

Protein modelling

Protein structure homology modelling was performed using the SWISS-MODEL workspace (Biasini*, et al* 2014). The SWISS-MODEL template library (SMTL version 2017-02-01, PDB release 2017-01-26) was searched with Blast and HHBlits for evolutionary related structures matching the target sequence. Results are shown using the myosin 2 heavy chain template (amino acids 1-959). There is no model from amino acid 960 to 1960 due to the lack of homology with any other protein.

DNA extraction and Sanger sequencing

Peripheral venous blood was collected at over 100 hospitals by Hematologists with an interest in inherited BPDs and sent to the Cambridge Translational GenOmics (CATGO) laboratory in Cambridge (UK) in EDTA tubes. Genomic DNA was extracted and quantified using PicoGreen (ThermoFisher Scientific Inc., MA, USA). All variants described in this paper were confirmed by Sanger sequencing. Primers were designed using PRIMER3 and the amplification carried out in a GeneAmp PCR System 9700 using 10ng DNA. Amplicons were purified by using QIAGEN (QIAGEN, Venlo, The Netherlands) columns and sent for Sanger sequencing to Source BioScience (Source BioScience, UK). Ethics authorities and approval numbers are provided in **Table S1.**

Immunofluorescence

Immunofluorescence analysis was performed by two independent centres. In center 1, blood smears were fixed and permeabilized with ice cold acetone for 3 minutes, blocked with 1% BSA for 1 hour and incubated with a rabbit-anti human NMMHCIIA antibody (Sigma Aldrich, M8064) and an Alexa-Fluor® 488 conjugated secondary antibody (Life Technologies). Nuclei were stained with Hoechst. Specimens were mounted with the

ProLong Antifade medium (Molecular Probes) and analyzed at room temperature by a Carl Zeiss Axio Observer.A1 fluorescence microscope (Carl Zeiss Inc, Oberkochen, Germany).

In centre 2, blood smears were fixed with acetone for 2 min and blocked with goat serum donor herd (Sigma G-6767, St. Louis, US) followed by staining with an anti-NMMHCIIA (Sigma Aldrich M8064 St. Louis, US) followed by Alexa Fluor 488 (Thermofisher, Waltham, US). Fluorescence micrographs were acquired on Leica TCS SP5 (Leica, Wetzlar, Germany) laser scanning confocal microscope with a HCX PL APO 40.0x N.A.1.25 oil immersion objective. Alexa Fluor 488 was excited by a 561nm DPSS laser. Pinhole was set to 67.9 µm (1 Airy Unit) and emission was collected by PMT 2 (508nm - 540nm). Image processing and analysis was performed on Bitplane Imaris version 7.6.5 (Bitplane, Switzerland).

Gene expression analysis

BLUEPRINT RNA sequencing data (http://dcc.blueprint-epigenome.eu/#/datasets) were downloaded from EGA and processed as follow:

Trim Galore 0.3.7 (http://www.bioinformatics.babraham.ac.uk/projects/trim_galore/) with parameters "-q 15 -s 3 --length 30 -e 0.05" was used to trim PCR and sequencing adapters off. Trimmed reads were then aligned to the Ensembl v.70 human transcriptome using Bowtie 1.0.1 (PMID: 19261174) with parameters "-a --best --strata -S -m 100 -X 500 --chunkmbs 256 --nofw --fr". MMSEQ 1.0.8a with default parameters[2,3] was used to quantify (gene and) transcript expression. Mmdiff with parameters "-fixalpha -p 0 -uhfrac 1.0" was used to summarise and normalize (gene and) transcript expression for cell type.

Sequencing and bioinformatics pipeline

Genome Sequencing was performed using the Illumina TruSeq DNA PCR-Free Sample preparation kit (Illumina, Inc.) and an Illumina HiSeq 2000 or HiSeq-X, generating a minimum coverage of 15x for approximately 95% of the genome (see also http://iovs.arvojournals.org/article.aspx?articleid=2503479). Targeted sequencing was performed using an Illumina HiSeq2500 or HiSeq4000 generating a minimum coverage

of 20x for approximately 99.99% of the targeted regions (with an average coverage of 833±193 reads for the MYH9 gene). Reads were aligned to the Genome Reference Consortium human genome build 37 (GRCh37) using the Isaac Genome Alignment Software (HAS2: http://support.illumina.com/sequencing/sequencing_software/hiseq-analysis-software-v2-0.html). Genome VCF files (https://www.ncbi.nlm.nih.gov/pmc/articles/PMC3137218/) were merged with Illumina AGG3 software (https://github.com/Illumina/agg). Structural Variants (SVs) were identified in the individuals who had genome sequencing using two independent algorithms: Isaac Copy Number Variant Caller (Canvas, Illumina), which identifies copy number gains and deletions based on read depth, and Isaac Structural Variant Caller (Manta, Illumina), which identifies translocations, deletions, tandem duplications, insertions, and inversions based on both paired read fragment spanning and split read evidence.

References

Biasini, M., Bienert, S., Waterhouse, A., Arnold, K., Studer, G., Schmidt, T., Kiefer, F., Gallo Cassarino, T., Bertoni, M., Bordoli, L. & Schwede, T. (2014) SWISS-MODEL: modelling protein tertiary and quaternary structure using evolutionary information. *Nucleic Acids Res,* **42,** W252-258.

Supplemental Tables

Table S1. Ethics authorities and approval numbers

Name of national ethics authority responsible	Ethics approval number	Country
Cambridgeshire 1 Research Ethics Committee	10/H0304/66	UK
Ethics Committee of the University Hospital Leuven	ML3580	Belgium
Children's Hospital of Philadelphia Institutional Review Board	IRB#12-008603	USA
Beth Israel Deaconess Medical Center IRB	Protocol #: 2011P000337	USA
Ethics Board of the University of Perugia	2014-031	Italy

Table S2. Patients excluded from this study. BPD= bleeding and platelet disorders; MPV= mean platelet volume

Patient	Transcript NM_002473.5 Protein NP_002464.1	Reason of exclusion
51	c.906C>G p.(Phe302Leu)	Abnormal platelet function and abnormal dense granules. Normal platelet volume (MPV: 8.8 fl), platelet count 358x10^9/L.
52	c.1724G>T p.(Gly575Cys)	Cornelia De Lange Syndrome. Normal platelet volume (MPV:7.6 fl).
53	c.1747G>A p.(Asp578Tyr)	Normal platelet volume (MPV: 10 fl).
54	c.2344G>A p.(Val782Ile)	Ehler-Danlos syndrome. Normal platelet volume, MPV: 10.3 fl, platelet count 203x10^9/L. This variant is also present in one non-BPD patient.
55	c.2403G>T p.(Lys801Asn)	This case was explained with the presence of a pathogenic variant in another gene.
56	c.2708G>A p.(Arg903Gln)	Ehler-Danlos syndrome. Normal platelet volume (MPV: 8.3 fl), platelet count 710x10^9/L.
57	c.2912C>T p.(Ala971Val)	Platelet storage pool disease, normal platelet volume (MPV: 10.2 fl), platelet count 262x10^9/L.
58	c.2965A>C p.(Lys989Gln)	This variant is present in one non-BPD patient and absent in the affected mother. Platelet count 217x10^9/L.
59/60/61	c.3340T>C p.(Ser1114Pro)	This variant is present in five non-BPD patients.
62	c.3677G>A p.(Arg1226Gln)	This case was explained with the presence of a pathogenic variant in another gene.

63	c.3817G>A p.(Asp1273Asn)	This variant is present in three non-BPD patients.
64/65/66/67/68	c.4225G>A p.(Asp1409Asn)	This variant is present in 15 non-BPD patients. Normal platelet volume (MPV:8 fl).
69	c.4330A>C p.(Lys1444Gln)	Normal platelet volume (MPV: 7.2 fl).
70	c.4439C>G p.(Ser1480Trp)	Platelet function disorder. Normal platelet volume.
71	c.4529T>G p.(Met1510Arg)	Patient has macrothrombocytopenia, however this variant is present in the healthy mother. Negative for MYH9 aggregates at immunofluorescence analysis and with low conservation score.
72	c.4774C>T p.(Arg1592Trp)	This case was explained with the presence of a pathogenic variant in another gene.
73	c.4955G>A p.(Arg1652His)	Normal platelet volume (MPV: 7.7 fl).
74	c.5328C>A p.(Asn1776Lys)	Thrombotic patient with thrombocytosis.

Table S3. Centralized analysis of blood smears for the identification of Döhle-like inclusion bodies in patients with previously not performed or negative detection of Dohle-like inclusion bodies. Patients not listed in the table had the Döhle-like bodies previously detected by MGG-staining. n/a= not available; VUS= Variant of Uncertain Significance; MGG= May-Grünwald-Giemsa; IF= Immunofluorescence. * = represents the 18 patients available for the centralized analysis.

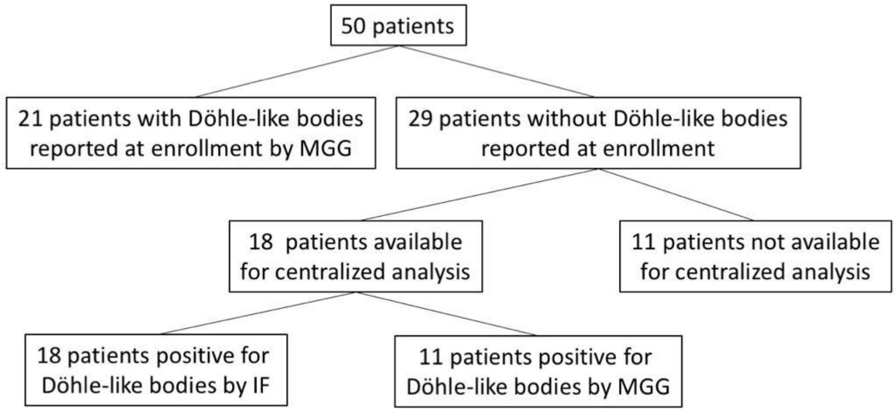

Patient	Transcript NM_002473.5 Protein NP_002464.1	Variant classification	Detection of Dohle like bodies from the submitting center	Result from centralized analysis (MGG)	Result from centralized analysis (IF)	Type of NMMIIA inclusion
**1***	c.97T>G p.(Trp33Gly)	Likely pathogenic	absent	present	present	II
**5**	c.279C>G p.(Asn93Lys)	Pathogenic	absent	n/a	n/a	n/a
**6***	c.283G>A p.(Ala95Thr)	Pathogenic	absent	present	present	III
**7***	c.287C>T p.(Ser96Leu)	Pathogenic	absent	uncertain	present	III
**9**	c.287C>T p.(Ser96Leu)	Pathogenic	absent	n/a	n/a	n/a
**10***	c.287C>T p.(Ser96Leu)	Pathogenic	absent	uncertain	present	III

**11***	c.1119G>C p.(Lys373 Asn)	Likely pathogenic	uncertain	present	present	II
**12**	c.2104C>T p. (Arg702Cys)	Pathogenic	absent	n/a	n/a	n/a
**13**	c.2104C>T p.(Arg702Cys)	Pathogenic	absent	n/a	n/a	n/a
**14***	c.2152C>T p.(Arg718Trp)	Pathogenic	absent	uncertain	present	III
**15***	c.2152C>T p.(Arg718Trp)	Pathogenic	absent	uncertain	present	III
**16***	c.2152C>T p.(Arg718Trp)	Pathogenic	absent	present	present	III
**18***	c.2507C>T p.(Pro836Leu)	Likely pathogenic	absent	present	Poor quality sample	n/a
**19***	c.2507C>T p.(Pro836Leu)	Likely pathogenic	absent	present	present	III
**20***	c.2668delC p.(Gln890Argfs Ter)	VUS	absent	present	present	III
**22**	c.3493C>T p.(Arg1165Cys)	Pathogenic	absent	n/a	n/a	n/a
**23**	c.3493C>T p.(Arg1165Cys)	Pathogenic	absent	n/a	n/a	n/a
**24**	c.3493C>T p.(Arg1165Cys)	Pathogenic	absent	n/a	n/a	n/a
**26***	c.3493C>T p.(Arg1165Cys)	Pathogenic	absent	present	Poor quality sample	n/a
**27**	c.3493C>T	Pathogenic	absent	n/a	n/a	n/a

	p.(Arg1165Cys)					
**28***	c.3584C>T p.(Ser1195Leu)	VUS	absent	absent	present	III
**29***	c.4262A>C p.(Glu1421Ala)	VUS	absent	uncertain	present	III
**30***	c.4270G>A p.(Asp1424Asn)	Pathogenic	absent	present	present	II
**32***	c.4270G>A p.(Asp1424Asn)	Pathogenic	absent	present	present	II
**34**	c.4270G>T p.(Asp1424Tyr)	Pathogenic	absent	n/a	n/a	n/a
**35**	c.4270G>T p.(Asp1424Tyr)	Pathogenic	absent	n/a	n/a	n/a
**37**	c.4302G>C p.(Gln1434His)	VUS	absent	n/a	n/a	n/a
**39***	c.2761G>A and c.4946A>G p.(Glu921Ly) and p.(Asp1649Gly)	VUS	absent	present	Only MGG stained smear available	n/a
**40***	c.5032A>G p.(Met1678Val)	VUS	absent	uncertain	present	III

Table S4. Severity of thrombocytopenia. A) Distribution of platelet count measurements in the cohort. Y axis: number of patients; X axis: platelet count. **B) Platelet count per patient.** Patients were divided in three groups according to platelet count. Patients were numbered from 1 to 50. In brackets, the corresponding platelet count are reported. Underlined and in italic the two cases with no thrombocytopenia but large platelets. Both automated and microscopic platelet counts are shown.

A

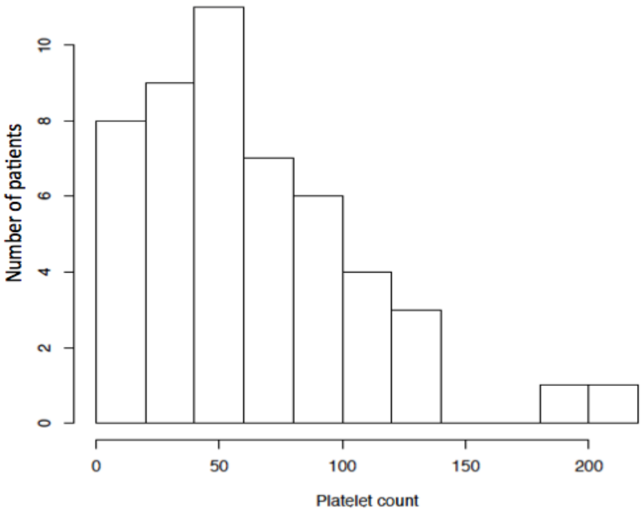

B

Platelet count (x10⁹/L)	< 20	20 - 50	>50
**Patient (automated platelet count)**	1(15); 4(8); 12(17); 33(11); 50(16)	2(46); 3(21); 5(21); 6(25); 8(21); 10(40); 11(45); 22^(46); 25(22); 28(20); 30(20); 32(37); 34∘(22); 36(47); 41(50); 42(20); 43″(50); 44″(47); 46(26);	7(121); 9(120) 13(74);14#(122); 15#(117); 16#(118); *17#(187);* 18(138); 19(81); 20(88); 21(70); 23(53); 24^(61); 26(62); 27(67); 29(60); 31(99); 35∘(61); 37(96); 38(68); 39(95); *40(220)*; 45(106); 47(59); 48(86); 49(54)
**Patient (optical platelet count)**	28(3)	3(25); 5(41); 43(50); 44(47)	2(80); 38(68); 48(86)

Members of the same family are identified with the following symbols: **#** (17 index case, 14 sister, 15 nephew, 16 mother) **^** (22 index case, 24 father), **∘** (34 index case, 35 mother)**,** **″** (44 index case, 43 daughter).

Table S5. Laboratory test results. Laboratory test results are displayed in this table. APTT = Activated Partial Thromboplastin time ; PT = Prothrombin Time; FBC = full blood count; RBC = red blood cell; HGB = haemoglobin; BASO = basophils; NEUT = neutrophils; LYMPH = lymphocytes; MPV = mean platelet volume; N/A = Not Applicable

Patient	Gender	Age at presentation (years old)	APTT (sec)	PT (sec)	FBC.RBC (x10e12/L); FBC.HGB (g/dl); FBC.BASO (x10e9/L);) FBC.NEUT (x10e9/L); FBC.LYMPH (x10e9/L); MPV (fL)
**1**	F	2	21.8	11.3	4.39; 14.5; 0.0; 3.0; 1.6; N/A
**2**	M	31	N/A	N/A	N/A
**3**	M	14	24.1	11.3	N/A; N/A; N/A; N/A; N/A; 16
**4**	F	3	N/A	N/A	3.54; 10.3; 0.3; 6.15; N/A
**5**	M	30	N/A	N/A	N/A; N/A; N/A; N/A; N/A; 14.5
**6**	M	2	N/A	N/A	4.48; 12.4; 0; 4.4; 6.2; N/A
**7**	M	N/A	N/A	N/A	N/A; N/A; N/A; N/A; N/A; 14.1
**8**	F	60	N/A	N/A	4.77; 14.2; 0; 8.6; 2.4; N/A
**9**	F	0	N/A	N/A	N/A; 12.2; N/A; N/A; N/A; 10.1 (note from clinician: macrothombocytopenia on examination of the smear)
**10**	M	0	N/A	N/A	4.84; 13.9; N/A; N/A; N/A; N/A
**11**	F	56	N/A	N/A	N/A
**12**	F	7	N/A	N/A	4.17; 11.6; 0.1; 1.9; 3.7; N/A
**13**	F	11	29.8	10	4.8; 13.5; 0.03; 6.5; 1.3; 10 (note from clinician: macrothombocytopenia on examination of the smear)
**14**	F	54	29.3	10.8	4.74; 14.4; 0.03; 3.24; 1.92; 15.1
**15**	F	N/A	N/A	N/A	4.67; 13.6; 0.03; 2.7; 2.21; 16
**16**	F	N/A	N/A	N/A	4.89; 15.4; 0.03; 8.15; 0.84; 15.1
**17**	F	N/A	N/A	N/A	4.64; 13.6; 0.07; 3.24; 1.79; 14.6
**18**	F	33	N/A	N/A	N/A;N/A;N/A;N/A;N/A; 14.5

**19**	M	38	N/A	N/A	N/A
**20**	M	86	25.8	12.3	4.3; 13.5; 0.03; 4.34; 0.8; N/A
**21**	F	5	N/A	N/A	N/A
**22**	M	21	N/A	N/A	5.24; 15.3; 0.02; 1.97; 1.48; N/A
**23**	F	N/A	N/A	N/A	N/A
**24**	M	45	N/A	N/A	5.18; 15; 0.04; 3.65; 1.27; N/A
**25**	F	31	27.8	9.7	4.3; 12.4; 0; 2.9; 0.8; N/A
**26**	M	58	26.2	10.2	4.62; 14.4; 0.05; 2.01; 1.20; 20.2
**27**	F	19	30.5	11.9	4.65; 14.5; 0.06; 3.33; 2.37; N/A
**28**	M	2	N/A	N/A	4.3;12;N/A;N/A;N/A;not detected by electronic counter
**29**	M	N/A	N/A	N/A	N/A
**30**	M	19	34.8	12.3	5.29; 13.6; 0; 1.8; 1.2; 8.2 (note from clinician: 8.2 is the automated count, macrothombocytopenia on examination of the smear)
**31**	M	2	N/A	N/A	4.44; 12; 0.1; 5.1; 6.3; 13.6
**32**	M	10	N/A	N/A	N/A; 15.1; N/A; N/A; N/A; N/A
**33**	F	N/A	N/A	N/A	4.47; 11.2; N/A; N/A; N/A; 11
**34**	F	22	19.7	10.1	6.0; 14.6; 0.03; 3.14; 1.03; N/A
**35**	F	43	26.4	12.3	4.98; 13.9; 0.07; 4.03; 1.95; N/A
**36**	F	27	N/A	N/A	N/A
**37**	F	N/A	24	10.6	5.47; 11.3; N/A; N/A; N/A; N/A
**38**	M	0	24.2	12.1	5.43; 14.6; 0; 7.5; 4; 13.8
**39**	M	30	26.3	10.1	5.48; 15.7; 0.03; 2.6; 1.56; 17.3
**40**	F	15	26.9	10.5	4.97; 13.6; 0; 2.9; 1.9; 12.2
**41**	F	56	N/A	N/A	N/A
**42**	F	22	N/A	N/A	N/A
**43**	F	16	23.1	11.9	4.37;12.7; 0; 1.1;1.8; 12.8
**44**	F	48	25.6	12.3	4.28; 12.1; 0; 4.8; 3.1; 13.3

**45**	F	29	N/A	N/A	N/A
**46**	M	18	N/A	N/A	5.02; 14.8; 0; 1.5; 1.6; N/A
**47**	F	46	N/A	N/A	4.06; 12.3; 0.03; 2.78; N/A; N/A
**48**	M	20	23.2	12.2	3.85; 11.8; 0; 0.2; 2; 12.5
**49**	M	2	24.2	10.4	N/A; 11.5; 3.13; 0.03; 2.98; N/A
**50**	F	8	N/A	N/A	N/A

Table S6. Bleeding score. Patients were classified according to the 12 major symptoms within the MCMDM-1 VWD Bleeding Assessment Tool with scores from -1 to 4. Total bleeding score are shown for females and males. Bleeding score is considered normal if less than 3 and 5 for males and females, respectively.

Bleeding Phenotype score	-1	1	2	3	4
**CNS bleeding**					
**Cutaneous**		5, 6*, 7, 10, 12, 18, 21, 22, 23, 25*, 28, 41, 43, 44, 46*, 47, 48, 49	1, 9, 16, 37,38		
**Epistaxis**		1, 18, 22, 24, 26, 28, 33, 40, 41,42, 43, 44, 46*, 50	14, 15,	16, 21, 30, 47	
**From minor wounds**		1, 18, 22, 30, 43, 44, 45*	9, 47		
**Haemarthrosis**					
**Menorrhagia**		4, 8*, 43	1, 18, 37	13, 40	17, 21, 34, 47
**Muscle Haematomas**		24, 47			
**Oral cavity**		46*, 47	14		
**Postpartum haemorrhage**	13, 14, 16, 37, 47	44	17	40	
**Surgery or major trauma**	13, 14, 17, 41, 47	8*	2, 5	30	16, 20
**Tooth extraction**	13, 14, 15, 16, 17, 21, 24, 37	8, 30, 40, 44, 45	34, 38		

**Total Bleeding Score**	**BS=0**	**BS 1-5**	**BS≧6**
**Female cases**	11, 13, 27, 29, 35, 36	4, 8, 9, 12, 14, 15, 17, 18, 23, 25, 33, 37, 41, 42, 43, 44, 45, 50	1, 16, 21, 34, 40, 47
**General Bleeding Severity**	**BS=0**	**BS 1-3**	**BS≧4**
**Male cases**	3, 19, 31, 32, 39	2, 5, 6, 7, 10, 22, 24, 26, 28, 46, 48, 49	20**, 30**, 38

* 6, 8, 25, 45, 46 are patients with no calculated bleeding score. The bleeding score has been assigned according to the clinical synopsis provided.

**prolonged bleeding after surgery

Table S7. Hearing impairment, renal and liver dysfunctions screening.
nv= normal values

Patient	Hearing impairment	Renal dysfunction	Liver
**8**	Complete deafness (the patient only communicates via sign language)	Mild proteinuria. Total protein loss in urine/24 hour: 2.06 g/24 h (nv <= 0.15 g/24 h); albumine/creatine ratio: 1520 mg/g creatinine (nv < = 30); microalbumine 506 mg/L (nl <= 20); microalbumine 1772 mg/24 h (nv <=30)	No dysfunction
**10**	Bilateral mild to moderate neurosensorial hearing loss (audiometric examination included tonal audiometry, logo-audiometry and tympanometry).	Mild proteinuria. Total protein loss in urine/24 hours: 0.3 g/24h (nv <= 0.15g/24 h). Serum creatinine: 0.62 mg/dL (normal range for age: 0.6–1.1 mg/dL); glomerular filtration rate (GFR): 80 mL/min/1.73m (normal range for age>75 mL/min/1.73 m).	No dysfunction
**12**	Clinically manifested hypoacusia	No dysfunction	No dysfunction
**17**	No dysfunction	*Renal anatomic abnormality (horseshoe kidney, not related to MYH9-RD). Normal albumin.*	No dysfunction
**18**	Mild high frequency sensorineural deafness. Audiograms: 60 dB at 4 kHz. Bilateral hearing aids used intermittently.	No dysfunction	Mildly elevated ALT (61-94 IU/L, (normal range 10-50). Bilirubin, ALP, albumin within normal limits.
**34**	Clinically manifested hypoacusia	No dysfunction	No dysfunction
**35**	Clinically manifested hypoacusia	No dysfunction	No dysfunction
**36**	Patient had full audiological work-up, demonstrating mild high frequency sensorineural hearing impairment. Bilateral hearing aids used intermittently.	No dysfunction	No dysfunction
**38**	Bilateral mild neurosensorial hearing loss for acute frequencies (audiograms).	No dysfunction	GTP=73 (normal values <45); GOT=53 (normal values<45); ALP =1880 (normal values<320); gamma-GT=36 (normal values <45)
**43**	Sensorineural drop right ear at 8KHz; normo-acusia left ear. No clinically manifested hypoacusia (audiograms).	No dysfunction	No dysfunction
**44**	Bilateral mild to moderate sensorineural hearing loss for acute frequencies (audiograms).	No dysfunction	No dysfunction
**48**	Severe bilateral sensorineural hearing loss for mid-high frequencies (audiograms).	No dysfunction	No dysfunction

Supplemental Figures

Figure S1. MYH9 protein structure model.

A. Protein structure homology model for MYH9. Results are shown using the myosin 2 heavy chain template (amino acids 1-959). From amino acid 960 to 1960 it is not possible to establish a model due to the lack of homology with any other protein. **B.** SH3/MD interface. **C**. In red, the amino acid in position 33 involved in the W33G variant is shown in the SH3/MD interface **D.** In red, the amino acid in position 41, involved in the F41S variant in the SH3/MD interface.

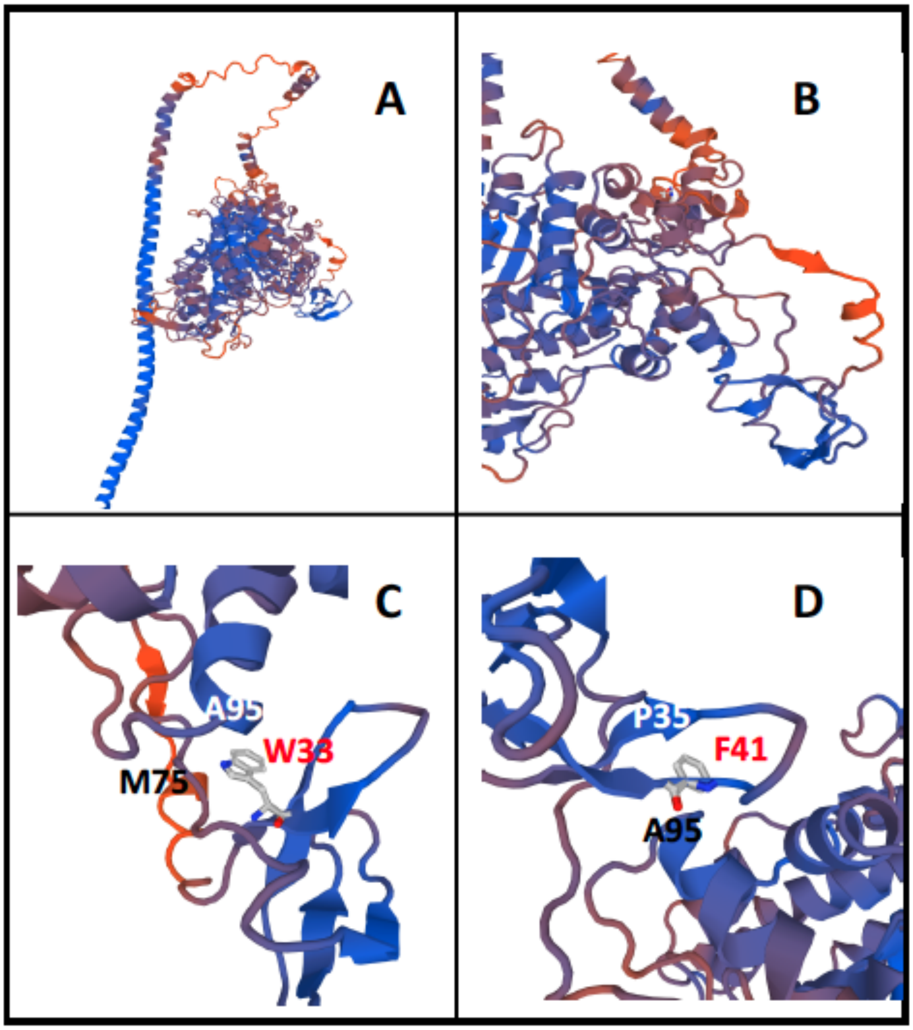

Figure S2. Coverage of the 12 novel variants. A) Whole Genome Sequencing (WGS) read coverage. Coverage plots for the five WGS cases. Case 39 has two novel variants. Mean read coverage 69X. **B) Targeted sequencing read coverage**. Coverage plots for the seven cases enrolled on the targeted ThromboGenomics platform. Mean read coverage 2300X. First and last exons highlighted.

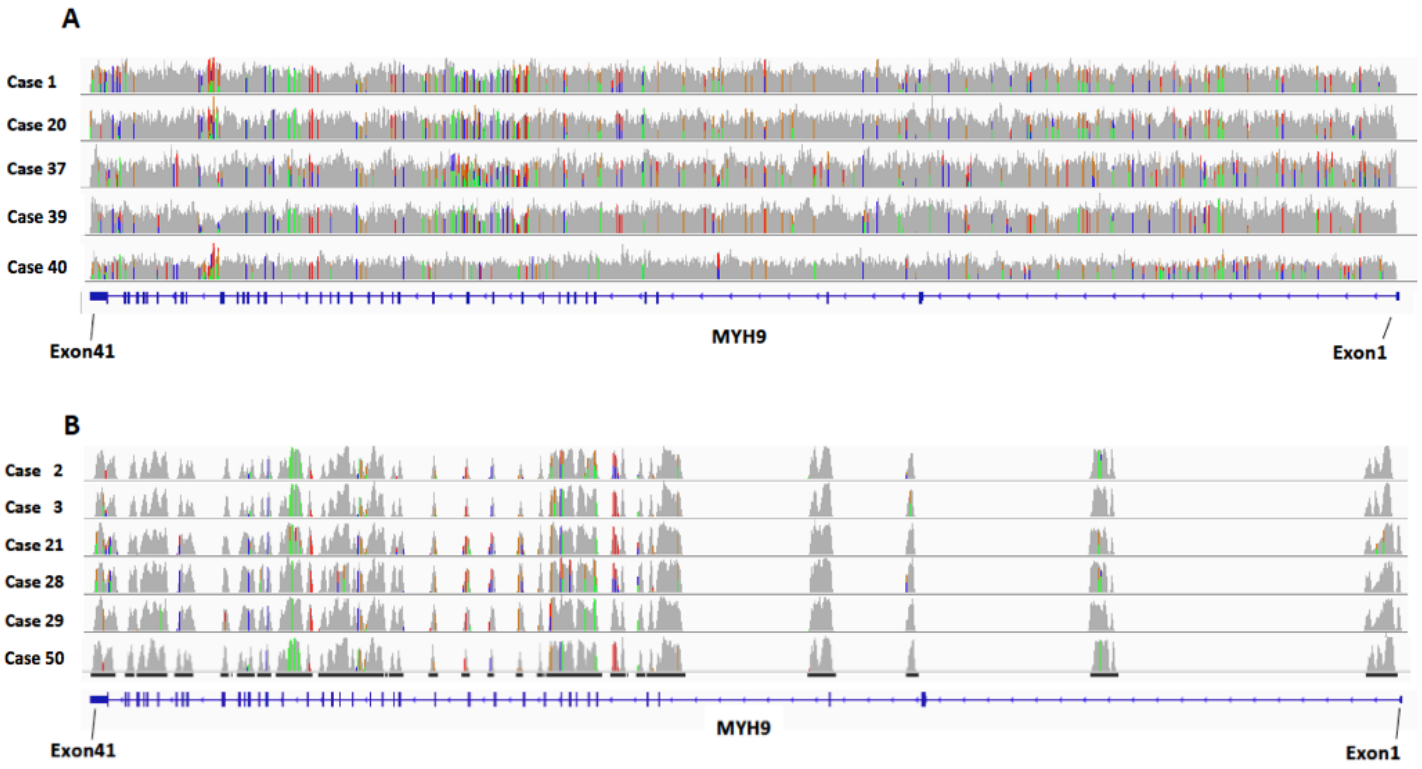

Figure S3. Expression levels of MYH9 transcripts across different blood cell types. Normalised expression of MYH9 transcripts in platelets (Plat), megakaryocytes (MK), erythroblasts (EB) and neutrophils (Neut) obtained from venous blood and cord blood, in dark and light green, respectively. Cell types are represented in brown for platelets, ochre for megakaryocytes, pale yellow for erythroblasts and grey for neutrophils. The top three rows show the expression level of the protein coding transcripts (rectangles with red, pink and pale pink on the left of the main panel). In the same column, in grey, the non-coding protein transcripts. ENST00000216181 (NM_002473.5) is the full length transcript (7501 bp, 1960 aa). High relative expression is shown in red and low relative expression in blue according to $\log_2$(FPKM+1).

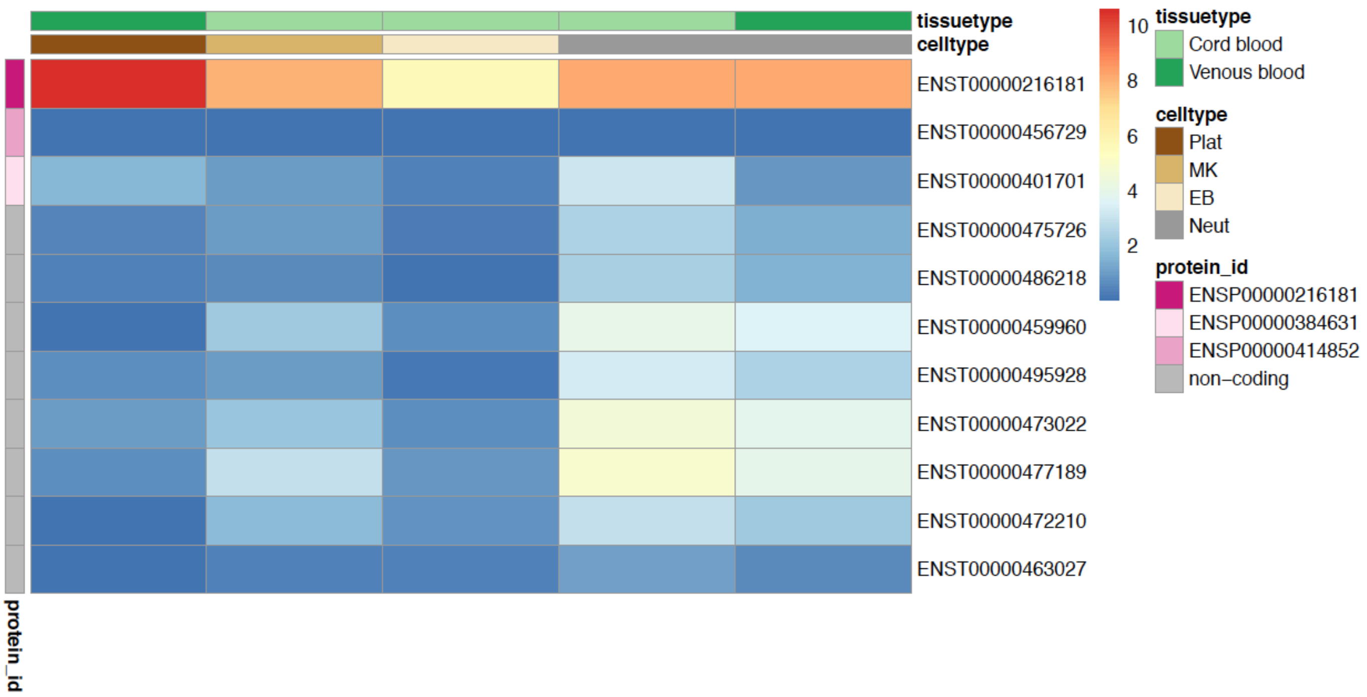

Figure S4. Phenotype-genotype correlation. The x axis indicates the exons of the *MYH9* gene with one or more variants found in this cohort and the y axis shows the number of patients with a specific phenotype. Patient 39 is represented twice due to the presence of two variants in exons 22 and 33. Patient 2 has been reported with a platelet count of 46x10^9/L and 80x10^9/L. In this graph patient 2 has been included to the group of patients with platelet count >50x10^9/L.

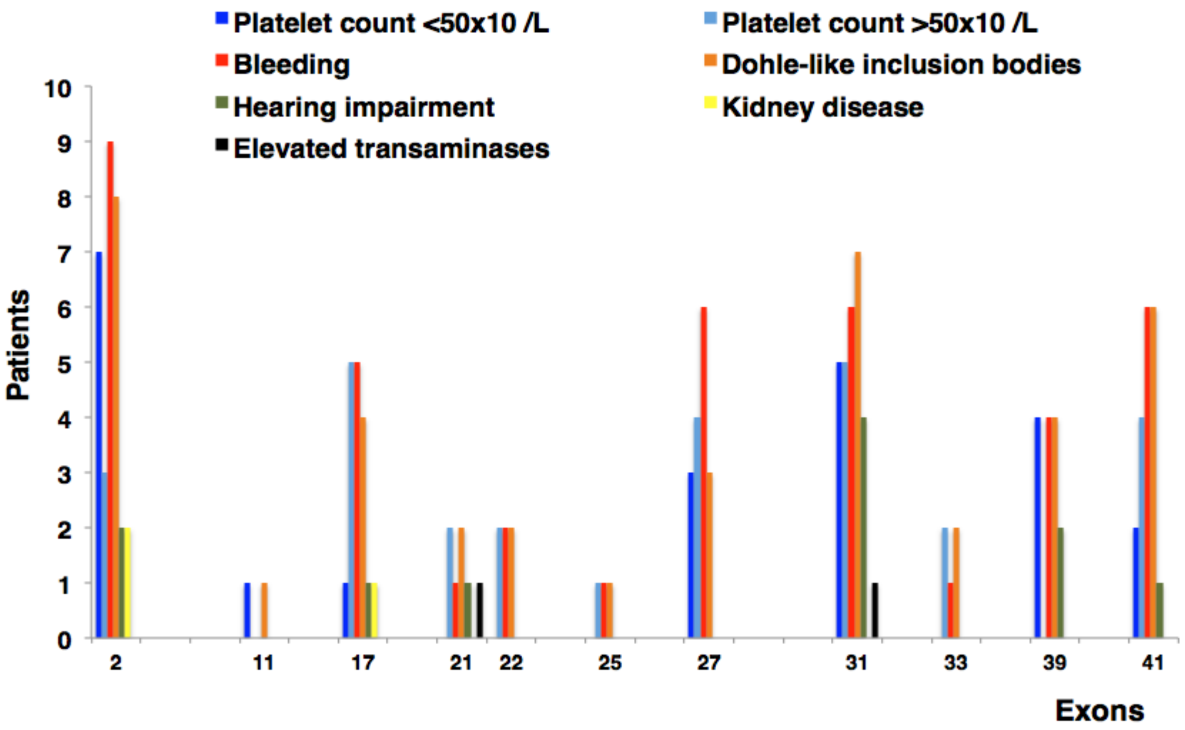

Figure S5. Distribution of bleeding tendency and platelet count. Patients have been divided in three groups according to their bleeding scores: no bleeding (BS=0), normal bleeding (BS= 1-5 for female or BS= 1-3 for male), excessive bleeding (BS>6 for female or BS>4 for male). No significant correlation has been found. Further details on bleeding score calculation can be found in Materials and Methods. BS= Bleeding Score; square= male patient; circle= female patient;

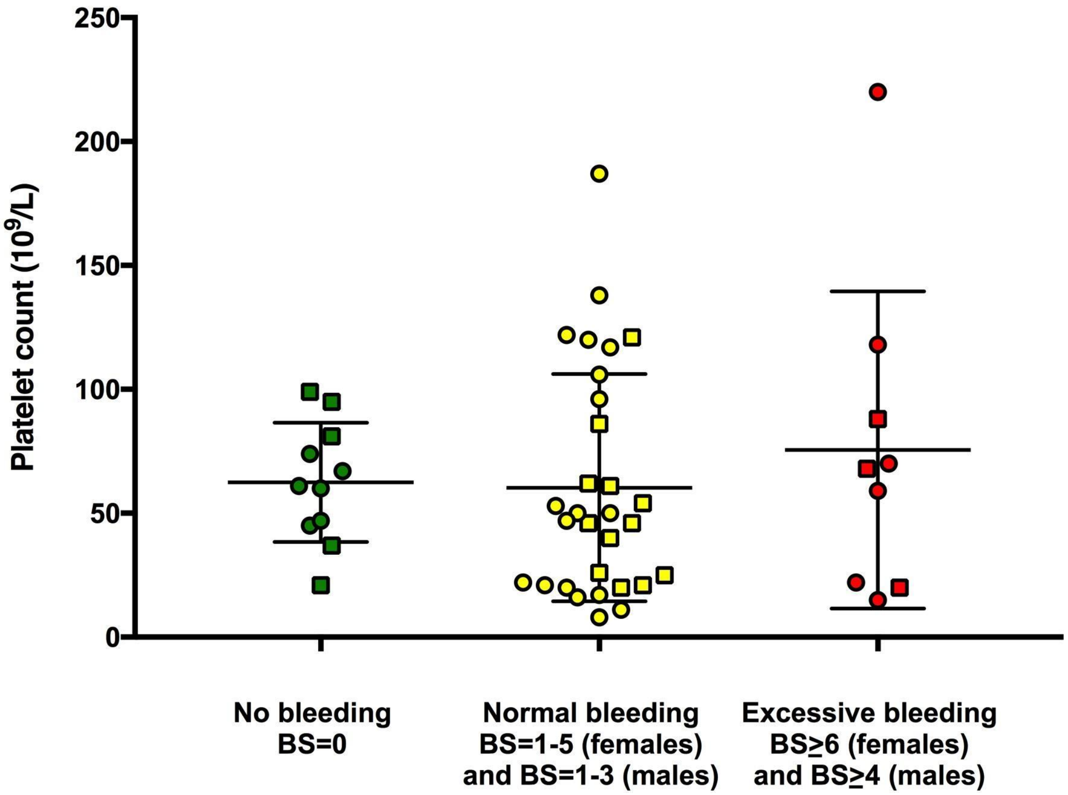

Figure S6. NMMHC-IIA localization and Döhle-like inclusion bodies analysis for the p.Gln890ArgfsTer variant. Light microscopy (**A**) and immunofluorescence (**B**) analyses of granulocytes in patient 20 show a non classical distribution of NMM-IIA, with some small punctuate clusters.

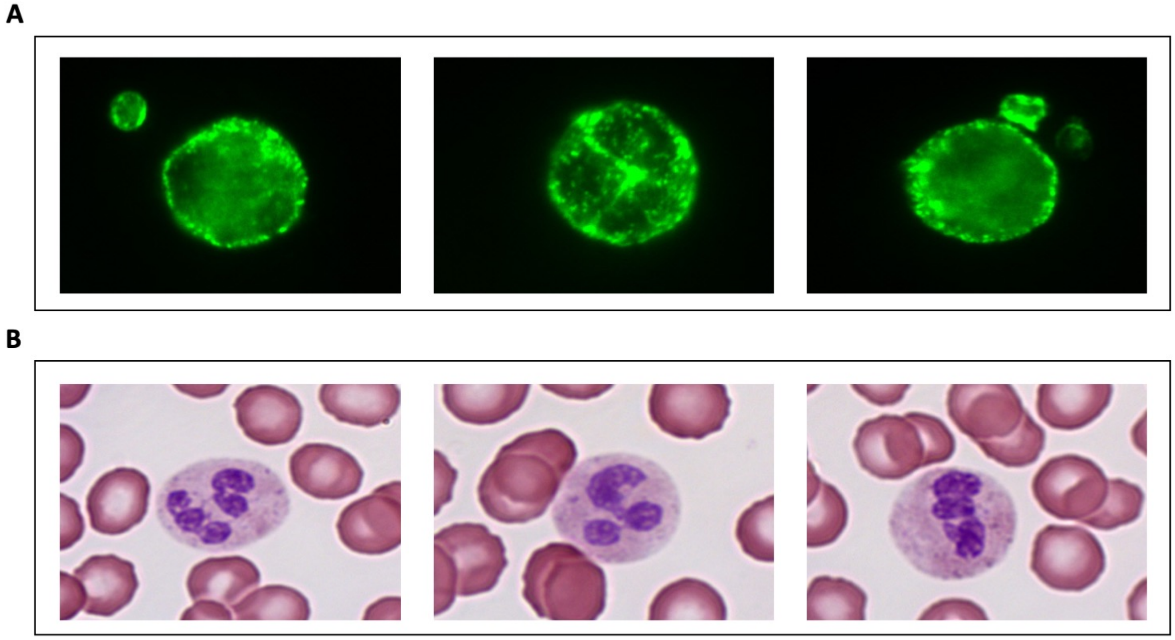

Supplement: Supplementary file 1 — Supporting information [file HUMU-41-277-s001.pdf]
